# Supplementary material for: Automated high-throughput genome editing platform with an AI learning in situ prediction model
Source: Nat Commun. 2022 Nov 30;13:7386. doi: 10.1038/s41467-022-35056-0 (PMC9712529; doi:10.1038/s41467-022-35056-0)
Supplement: Supplementary file 1 — Supplementary information [file 41467_2022_35056_MOESM1_ESM.pdf]

Supplementary information for

Automated high-throughput genome editing platform with an AI learning *in situ* prediction model

Siwei Li et al.

Supplementary Fig. 1. Detailed workflow of each module of the automated gene editing in mammalian cells.

Supplementary Fig. 2. The inputs, outputs, and human intervention steps in modules of the automated mammalian cells gene editing platform.

Supplementary Fig. 3. Time and money costs comparison of each module of manual operation and throughput platform gene editing in mammalian cells.

Supplementary Fig. 4. Editing efficiency of different cell lines and CBE base editors *in situ* targets. All experiments were performed as mean ± SEM from 100 biologically independent cells experiments.

Supplementary Fig. 5. Building and assessment of chromatin accessibility enabled learning model (CAELM) via 5X5 nested cross validation.

Supplementary Fig. 6. The construction and correction of SNV cell models.

Supplementary Fig. 7. Monoclonal sorting strategy of SNV cell models by FACS.

Supplementary Fig. 8. ccdb-gRNA plasmid map and sequence.

Supplementary Fig. 9. BE4max plasmid map and sequence.

Supplementary Fig. 10. AncBE4max plasmid map and sequence.

Supplementary Fig. 11. hyA3A-BE4max plasmid map and sequence.

Supplementary Fig. 12. ABE8e plasmid map and sequence.

Supplementary Fig. 13. APOBEC3A-nCas9 plasmid map and sequence.

Supplementary Table 1. The sequences of the actual efficiencies for the high-throughput system and manual manipulation at 32 genomic loci.

Supplementary Table 2. The sequences of the actual efficiency of BE4max and APOBEC3A-nCas9 at 30 genomic loci.

Supplementary Table 3. The sequences information of endogenous targe sites at 10 genomic loci.

Supplementary Table 4. The approximate contribution of the chromosomal environment condition to the prediction relative to the contribution of the DNA sequence context to the different models including different cell lines and CBE base editor.

Supplementary Table 5. The nine pair primers of construction gRNAs for adenine base editor (ABE).

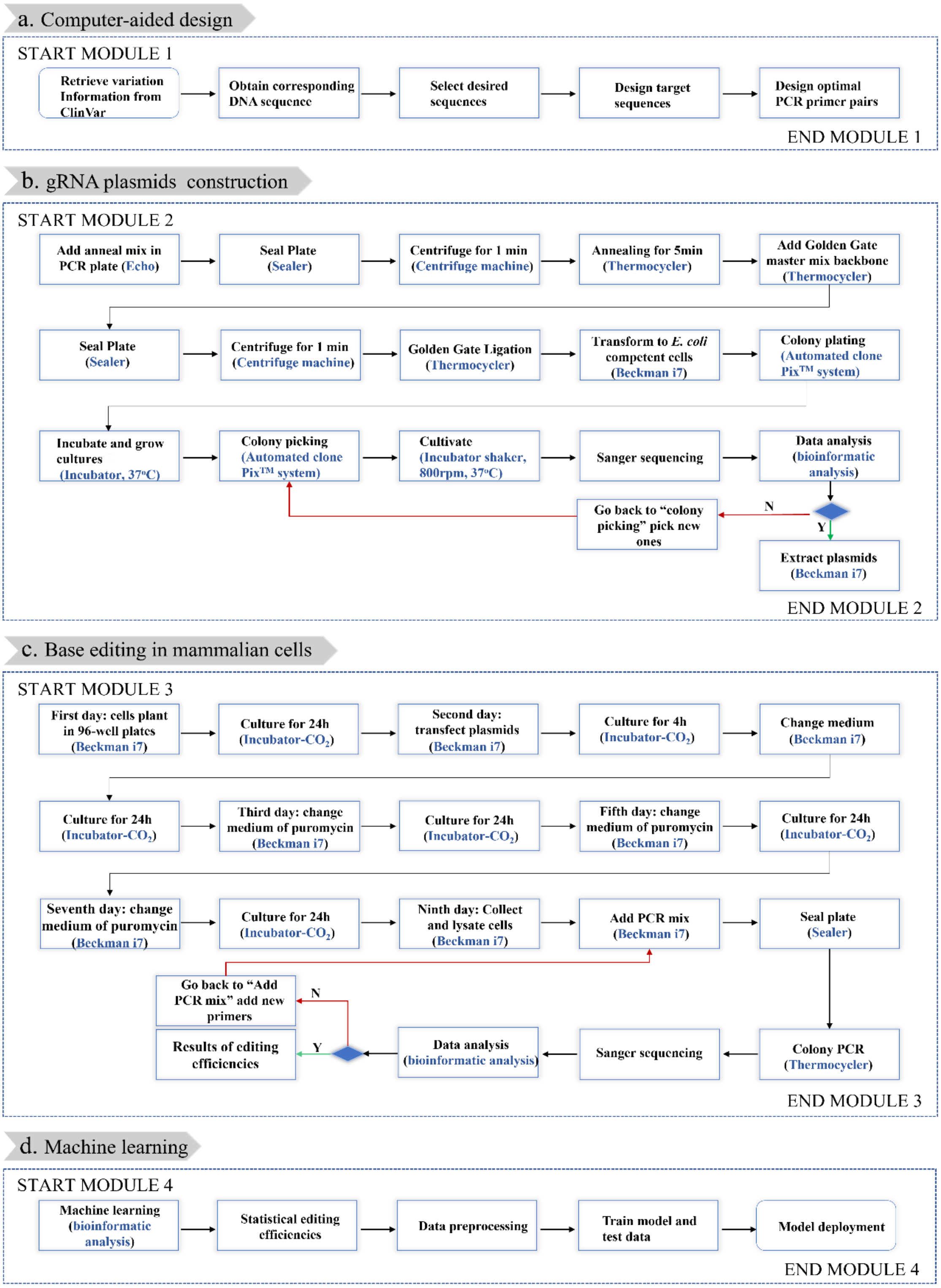

Supplementary Fig. 1. Detailed workflow of each module of the automated gene editing in mammalian cells. (a) Machine design module: gRNA plasmids design and batched primers acquisition. (b) gRNA plasmids construction module. (c) Cells base editing module. (d) Machine learning of the *in situ* editing results of cells. The red arrow means wrong results, and more colonies need to be picked or change the primers for verification. The green arrow means correct results and can be made downstream. The steps of experiments are listed in black character and the corresponding instrument are listed in blue.

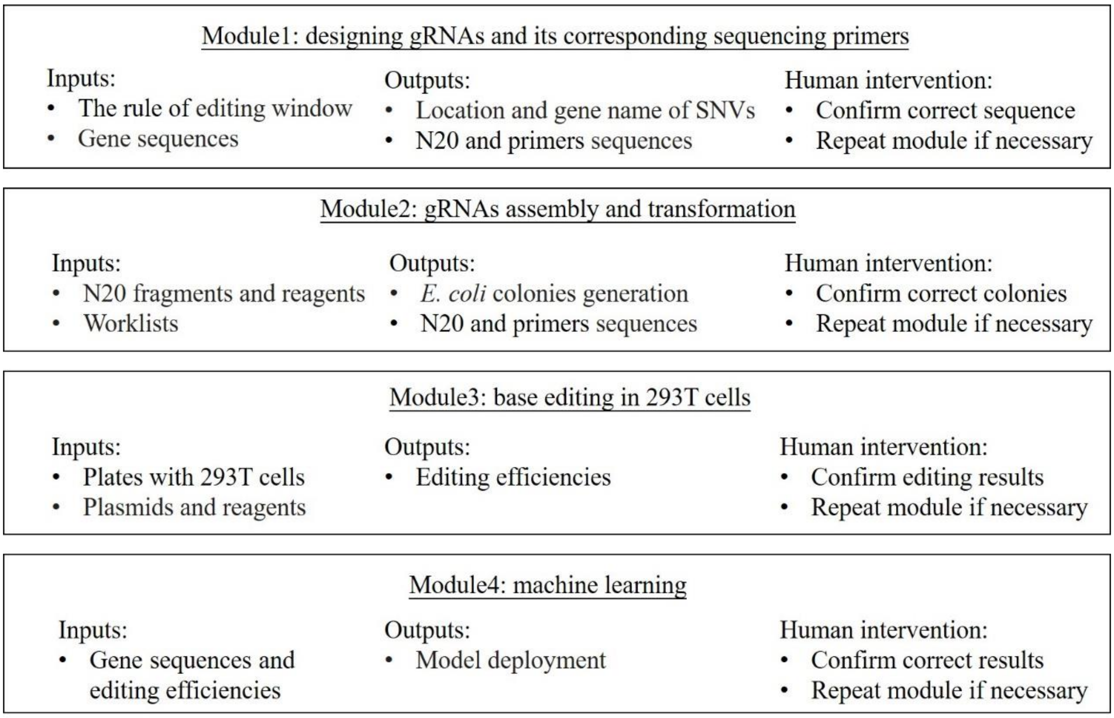

Supplementary Fig. 2. The inputs, outputs, and human intervention steps in modules of the automated mammalian cells gene editing platform.

a

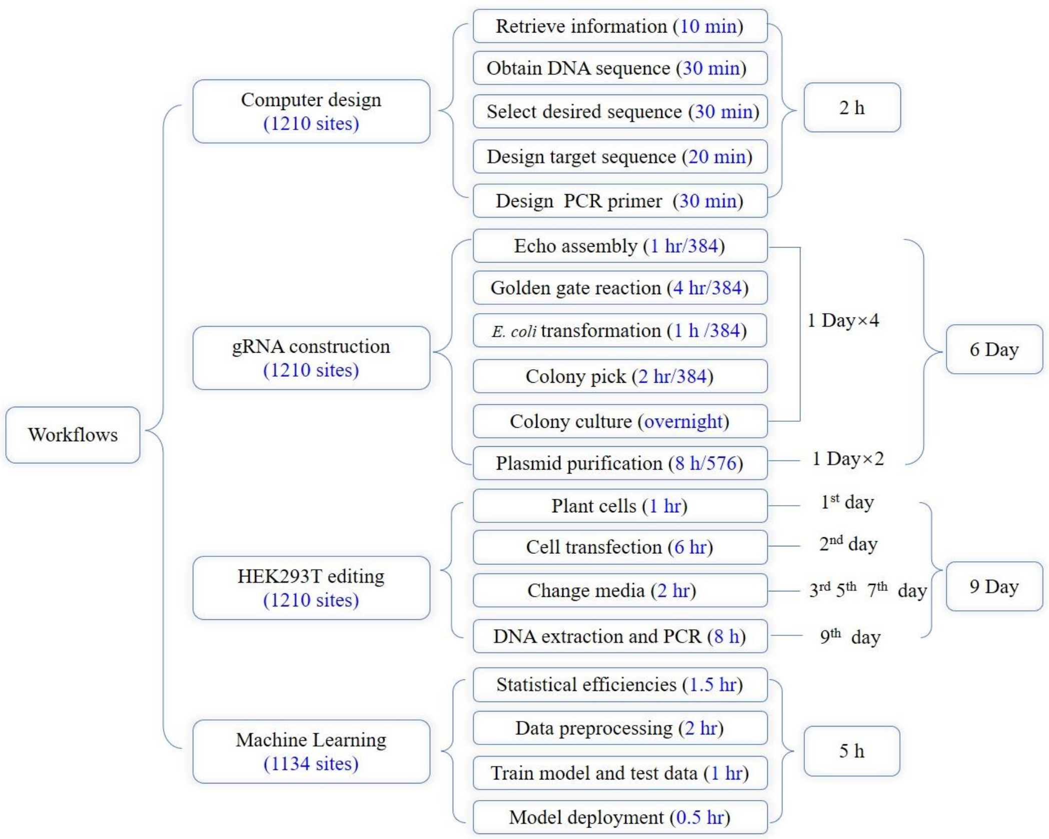

b

	Design		gRNA plasmids consturction		Base editng		Machine learning	
	Time	Cost	Time	Cost	Time	Cost	Time	Cost
Manual operation	30 d	$5.01/sample	17.6 d	$10.46/sample	7.04 d	$2.88/sample	7.04 d	$2.00/sample
Throughput platform	2 h	$0.036/sample	31 h	$9.01/sample	12.5 h	$2.25/sample	12.5 h	$0.07/sample

c

	**Machine Design**			
	Retrieve information	Obtain DNA and select desired sequence	Design target sequence	Design PCR primer
Manual operation	1 d	7 d	7 d	15d
Throughput platform	10 min	1 h	20 min	30 min
	**gRNA plasmids consturction**			
	PCR mix assembly	Golden gate reaction	*E. coli* transformation and pick	Plasmid purification
Manual operation	1 d	4 h	4 d	12 d
Throughput platform	3 h	4 h	8 h	16 h
	**Base editng**			
	Plant cells	Transfection	Change medium	DNA extraction and PCR
Manual operation	1 h	3 d	1 d	3 d
Throughput platform	0.5 h	3 h	1 h	8 h
	**Machine learning**			
	Statistical efficiencies	Data preprocessing	Train model and test data	Model deployment
Manual operation	10 d	2 d	1 h	0.5 h
Throughput platform	1.5 h	2 h	1 h	0.5 h

Supplementary Fig. 3. Time and money costs comparison of each module of manual operation and throughput platform gene editing in mammalian cells. (a) Time costs of process modules and total operations in the workflow of mammalian cells gene editing. (b) Time and money cost comparison of manual operation and throughput platform. (c) Detailed time comparison of each module of manual operation and throughput platform.

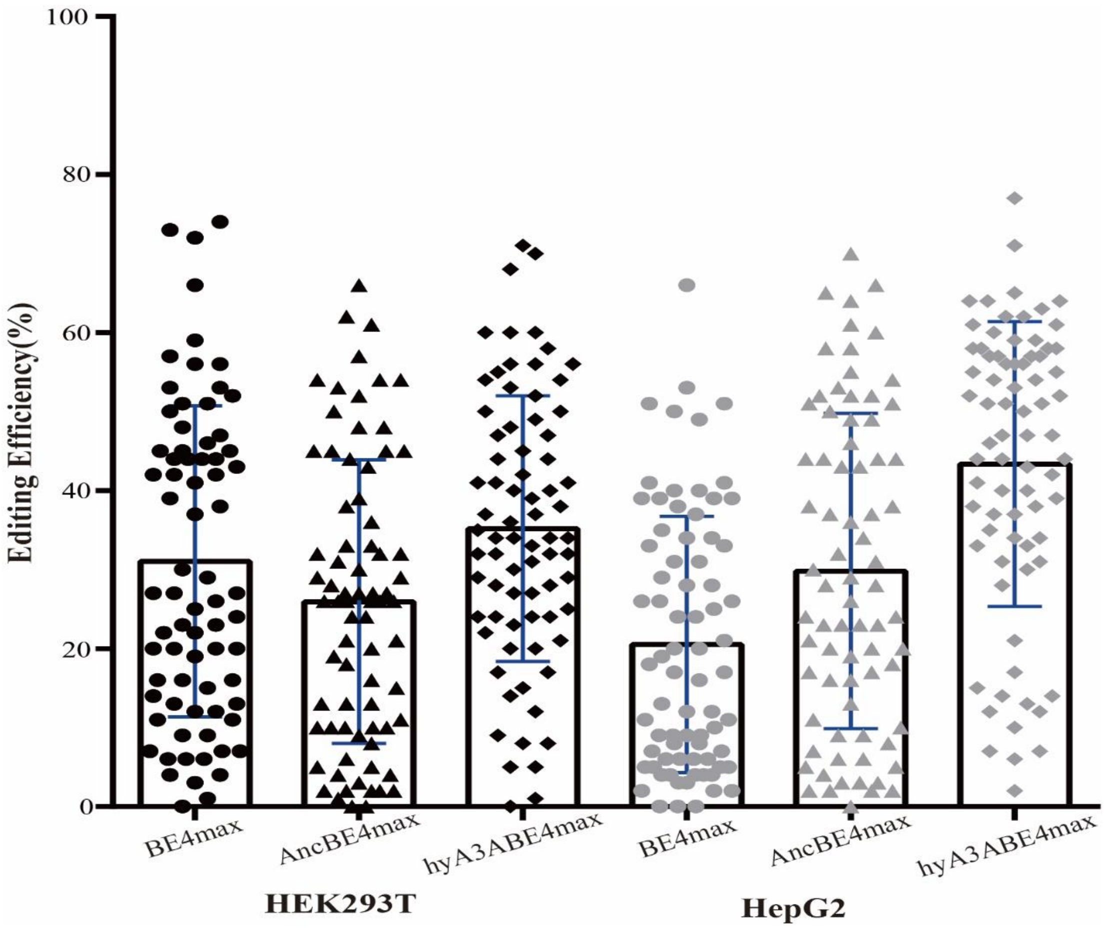

Supplementary Fig. 4. Editing efficiency of different cell lines and CBE base editors *in situ* targets. All experiments were performed as mean ± SEM from 100 biologically independent cells experiments.

a
b
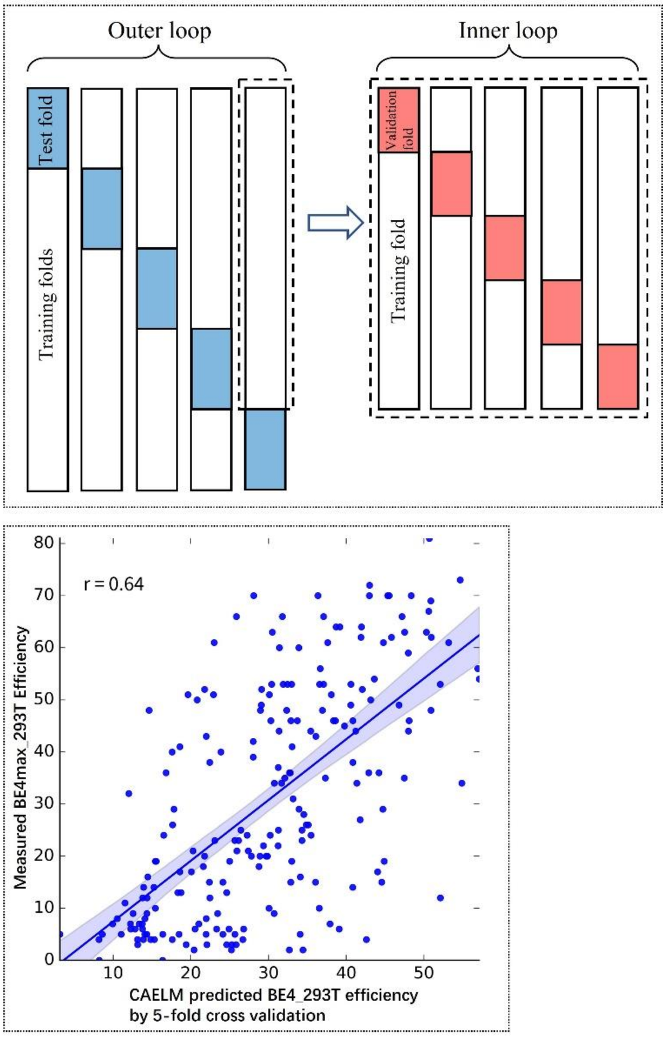

Supplementary Fig. 5. Building and assessment of chromatin accessibility enabled learning model (CAELM) via 5X5 nested cross validation. (a) Schematic diagram of the 5x5 nested cross validation. (b) Linear regression plot of CAELM to predict BE4max_293T efficiency (fit line and its confidence-interval band is presented).

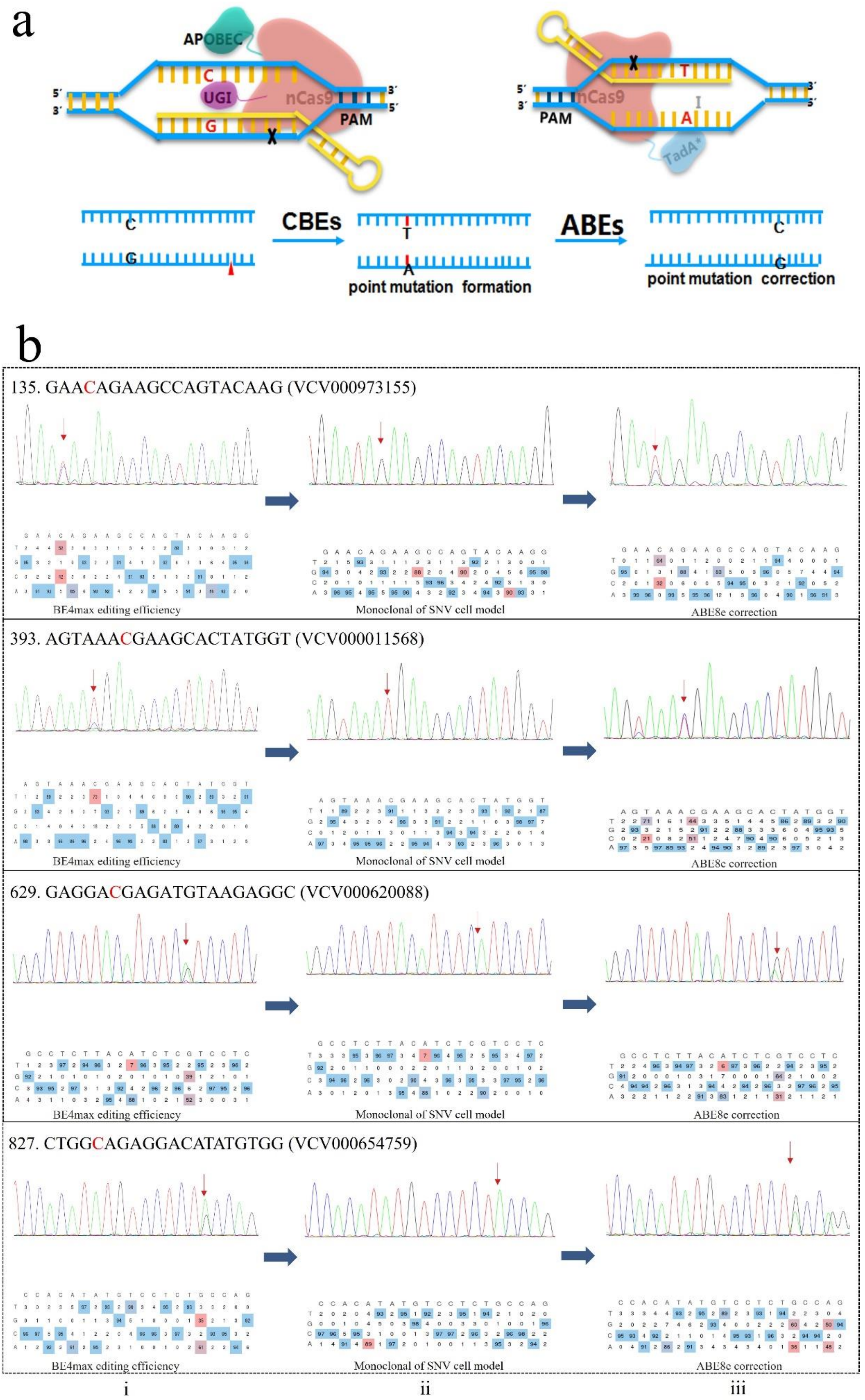
a
APOBEC
UGI
nCas9
PAM
5′
3′
C
G
X
T
A
I
TadA*
CBEs
ABEs
point mutation formation
point mutation correction
b
135. GAACAGAAGCCAGTACAAG (VCV000973155)
393. AGTAAACGAAGCACTATGGT (VCV000011568)
629. GAGGACGAGATGTAAGAGGC (VCV000620088)
827. CTGGCAGAGGACATATGTGG (VCV000654759)
BE4max editing efficiency
Monoclonal of SNV cell model
ABE8e correction
i
ii
iii

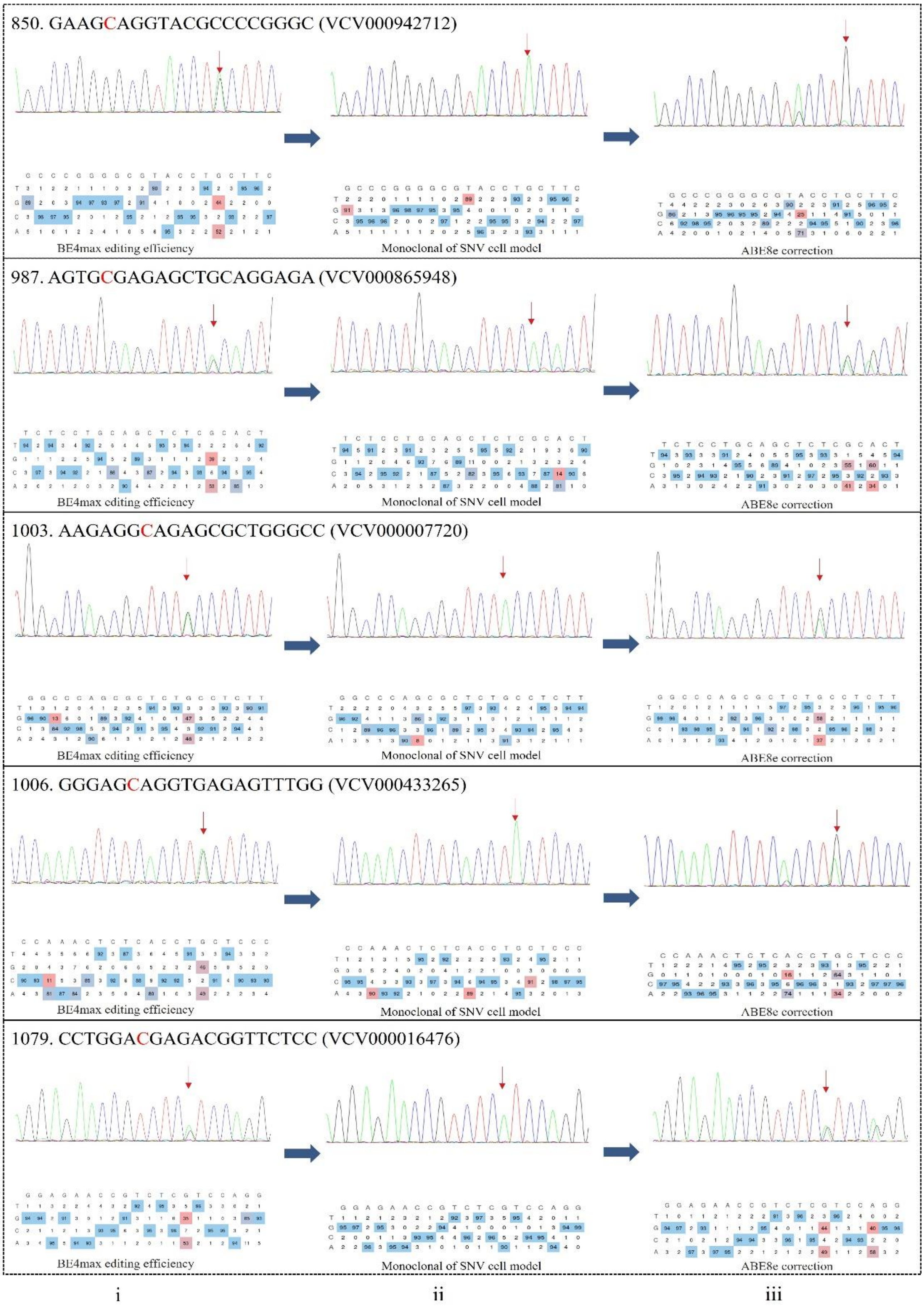

Supplementary Fig. 6. The construction and correction of SNV cell models. (a) The editing process of building disease models by BE4max and correction of SNV cell models by ABE8e. (b) i. 9 targets editing peak figures by BE4max editing; ii. monoclonal SNV cell models verify peak figures; iii. the correction peak figures of SNV cell models by ABE8e editing.

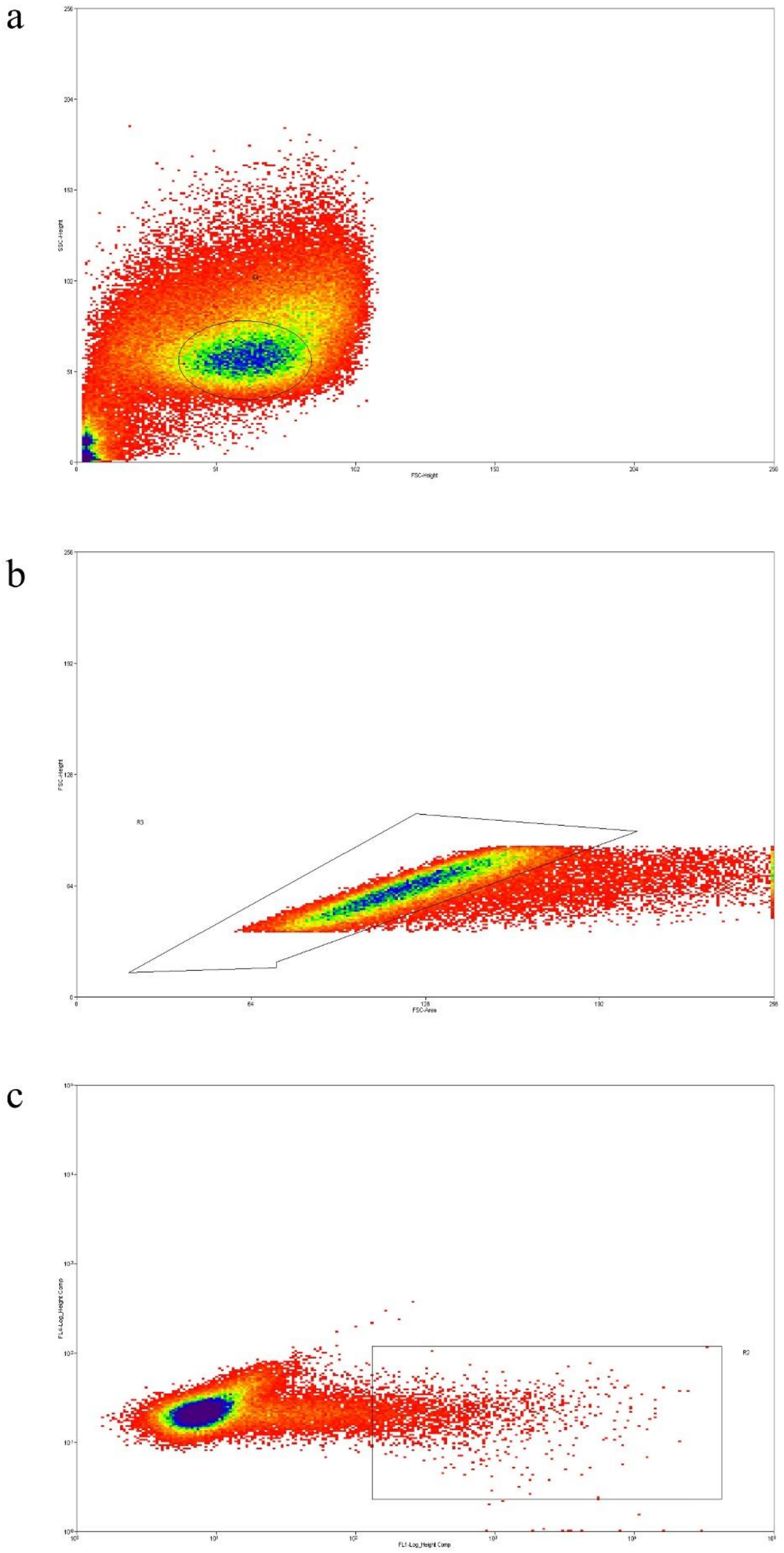

Supplementary Fig. 7. Monoclonal sorting strategy of SNV cell models by FACS. (a) FSC channel for setting. (b) FSC-H-FSC-A for remove the cell adhesion. (c) GFP mono-positive region for sorting.

Supplementary Table 1. The applied protospacer sequence and the mean values of the actual efficiencies for the high-throughput system and manual manipulation at 32 genomic loci.

Target sites	Sequence	High throughput	Manual operation
1	CTCGAGAAAGTGACCCTGTC	37	19
2	GCCATGGTGGACAGAGGGAC	19	20
4	GGGTGATTCTGGGAAGAGAA	42	38
7	CCCGGTGTAGAAGGTGATGT	34	24
9	GCTGCGAAGGGATTTGGGGG	16	19
12	ACATCTGAAACATGAAATGG	61	58
13	AAAGTGGCTAAAATTATAAA	13	7
16	TTTGGACAACCCTCTGTACC	38	24
17	AGAACAATTTGAAGGAAAGA	44	35
26	TTTAAACGAGTATTCCTAAA	4	4
31	CAGCGGATTTAGCTGCTCCT	3	4
41	CCTCGATGTGCTTTAGCCAC	69	56
44	AAAAAGCAGGTGAGTGCCAT	10	5
112	CGAGCGGGTGCACAAGGTCA	27	24
115	GCTCATAGGCTGCTGCCAGC	22	39
135	GAACAGAAGCCAGTACAAGG	52	52
262	ACCCTTTGGGAGGCAAACAG	8	8
495	CTCTTATATCTACAGTGTGG	80	59
499	TGTATACAGTCTTATTACAG	54	34
518	GAGAGTCGAGTAGTTTCTGC	78	51
545	CCTTCGATGGAGAGCCTTCG	54	55
556	GATGCGAGACAAATACAAAG	72	60
558	TGTGAACGATGTCCTACCTG	57	52
560	CATTTCGTTATCATCATCAG	82	65
900	CGGCAGGTAAGTTGACACCC	22	19
933	ACTTACTGAGAGCCATGCAA	31	59
936	GGAACTTGGAACAGAGACCT	47	58
937	CATGCGGGTGCTCCTGCTGG	41	39
954	AATGCAAGGAATGGATTCTG	48	44
956	ATCGAGTGGCTGACCAGCTA	43	30
958	TGACGAAGCTAATAGGAGAG	38	34
960	ATGGCTGAATAGATGCCACT	24	15

Supplementary Table 2. The applied protospacer sequences and the mean values of the actual efficiency of BE4max and APOBEC3A-nCas9 at 30 genomic loci.

targets sites	sequence	BE4max	A3A-nCas9
26	TTTAAACGAGTATTCCTAAA	4	33
31	CAGCGGATTTAGCTGCTCCT	3	36
54	GCGTAGAACATTGTTCTGGC	5	40
95	GAAGATGCAGCCAAGCATGA	4	42
123	TTTGTTGCAGTTATCCAAGA	4	52
170	TTAAACTGAGGAATGCCATC	4	34
195	TGAATTGCATATACATTTAT	6	32
212	TTGAGGCAGAAGATCAAAAT	2	44
247	AAGCAAGTACTTACATCAAT	1	53
262	ACCCTTTGGGAGGCAAACAG	8	54
418	GGAATCGAACCCCCCAAAGC	3	2
422	CCCAGAGGTCGTTGTGCAGG	0	22
434	CGCGAGGTGGACGGGGCGGT	1	26
445	GTCGGTGAGTCTTTGCTTAC	7	6
552	CACATTTTGGAAAATTTCCT	3	13
562	TTCAGTGTAAAAATTTTACA	4	18
578	TTGAAAAACGAGAAGTCAGC	2	20
593	GACTGGAATTAATATTCAGC	7	28
619	TATGTAACCATTCATTTTGA	4	10
655	AAATTTTACGACCTTCTTTT	2	4
658	TTGTGGCATTTTTCATTGGC	3	17
707	GCTGCAGGATGGTGAGGACG	1	3
708	TCACTGTATCTTTCTAAACT	0	15
795	ATTATGCGCACCATGTTTTC	1	4
884	TTCAATTTATTGACTGTGTC	5	36
887	TCCATGATTCATTTGTATCT	4	14
928	GAACGAATGGATTTGTTATC	3	21
952	ACTATGAACAGTTGCCTTAT	1	10
974	GCTGGCAGACTGTCATTGAT	0	5
1095	TCCTGCAGACTCAGGATGAC	0	1

Supplementary Table 3. The sequences information of endogenous targe sites at 10 genomic loci.

Endogenous sites	N20 sequence	chromatin accessibility avg.	Actual Efficiency	Our Model	BE-Hive
Endo_site1	TCTTACAGATAGCGAAACTG	0.040345	38.04%	51.94%	43.40%
Endo_site2	AGCAAACGAGCTGAAAGCCA	0.012415	32.49%	36.27%	60.70%
Endo_site3	AATCGGTTTGTGTGGGGTGG	0.038185	17.05%	40.26%	74.20%
Endo_site4	GGCTGAACTAAAGCCTCCAG	0.027225	40.41%	36.05%	55.00%
Endo_site5	GAAGGCTGAACAGCAGGGGT	0.051475	57.39%	43.69%	66.50%
Endo_site6	GTTCGTGATGACCAAAACCT	0.019385	28.49%	47.84%	74.10%
Endo_site7	TTCGGCGAGCTGGCCACTGG	0.133275	17.50%	53.63%	44.00%
Endo_site8	AGAGCAGGAAGAAATGCAAG	0.00732618	34.26%	35.20%	87.00%
Endo_site9	GAAACAGCTGATTCTAGGGT	0.048725	67.35%	54.30%	63.30%
Endo_site10	AAAATGATCAAATAACTAGG	0.04455	40.27%	37.99%	67.00%

Supplementary Table 4. The approximate contribution of the chromosomal environment condition of the prediction relative to the contribution of the DNA sequence context to the different models including different cell lines and CBE base editor.

Cell lines	BE4max	Anc-BE4max	hyA3A-BE4max
293T	0.140766098093608	0.14420118343195265	0.14420118343195265
HepG2	0.14728286620257316	0.14661393969352446	0.12905159067072866

Supplementary Table 5. The nine pair primers of construction gRNAs for adenine base editor (ABE)

Samples	PAM	Primer-F (5'-3')	Primer-R (5'-3')
135	CAT	CACCGTTCTATTCAG GTATCTGTTT	AAACAAACAGATACCTGAATAGAAC
393	AGG	CACCGTGC TTCATTTACT TTGCTC	AAACGAGCAAAGTAAATGAAGCAC
629	AGG	CACCGTCTCATCCTCGGGGGCCAAC	AAACGTTGGCCCCCGAGGATGAGAC
827	TGG	CACCGCCTCTACCAGGCCTCCAGGA	AAACTCCTGGAGGCCTGGTAGAGGC
850	TGG	CACCGACCTACTTCTGGTAAGGGTC	AAACGACCCTTACCAGAAGTAGGTC
987	CGG	CACCGCTCTCACACTGCCTCCTCC	AAAC GGAGGAGGCAGTGTGAGAGC
1003	TGG	CACCGCTCTACCTCTTCGACGTGGA	AAACTCCACGTCGAAGAGGTAGAGC
1006	TGG	CACCGCCTACTCCCCAATTGAAGTG	AAACCACTTCAATTGGGGAGTAGGC
1079	CGG	CACCGTCTCATCCAGGGGAACCTT	AAACAAGGTTCCCCTGGATGAGAC

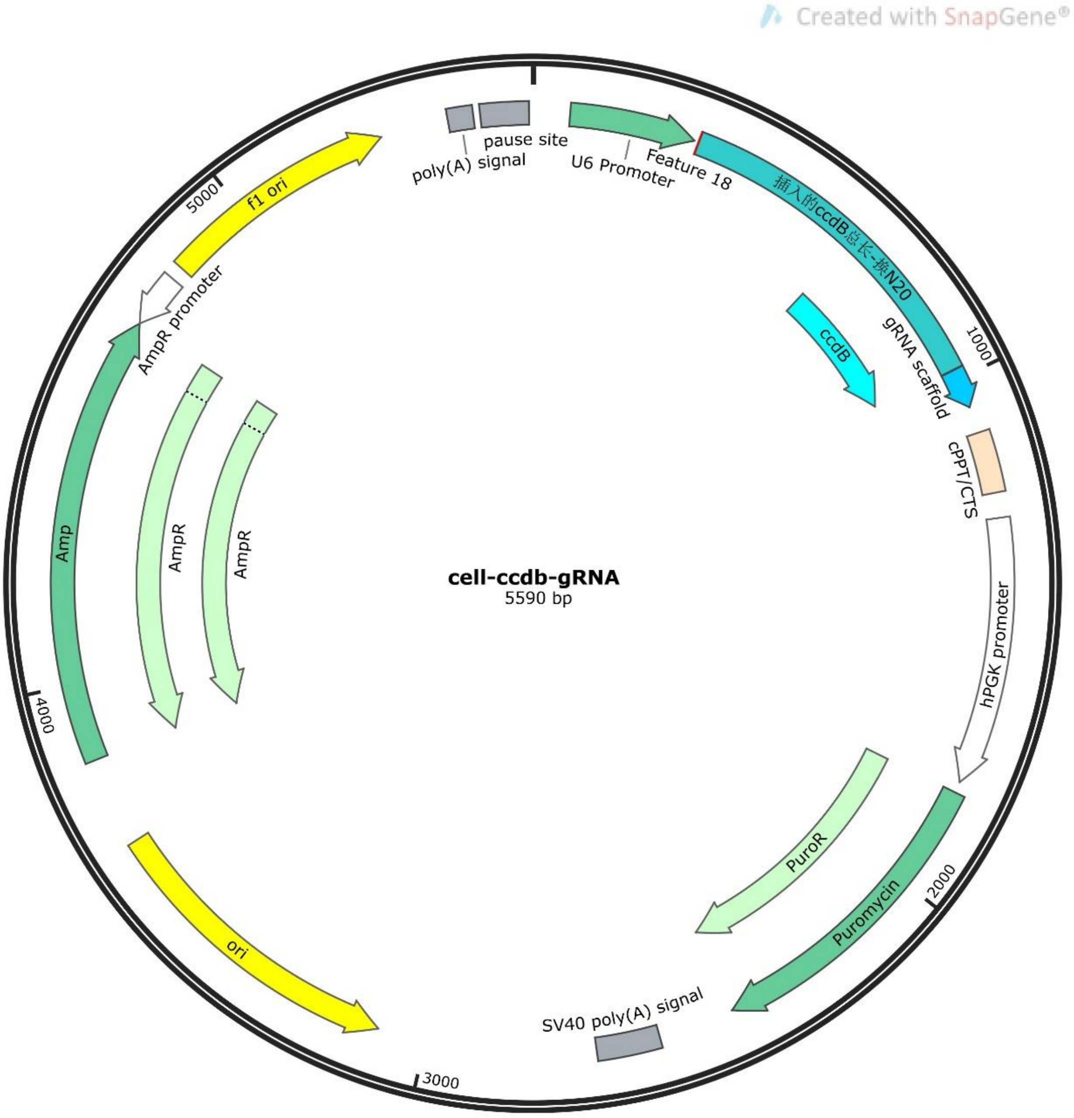

Supplementary Fig. 8 ccdb-gRNA plasmid map

Ccdb-gRNA plasmid sequence:

ggtaccgattagtgaacggatctcgacggtatcgatcacgagactagcctcgagcggccgccccttcaccgagggccta
tttcccatgattccttcatatttgcatatacgatacaaggctgttagagagataattggaattaatttgactgtaaacacaaagata
ttagtacaaaatacgtgacgtagaaagtaataatttcttgggtagtttgcagttttaaaattatgttttaaaatggactatcatatgc
ttaccgtaacttgaaagtatttcgatttcttggctttatatatcttgtggaaaggacgaaacaccGgagaccacgcgtggatcc
ggcttactaaaagccagataacagtatgcgtatttgcgcgctgatttttgcggtataagaatatatactgatatgtatacccgaa
gtatgtcaaaaagaggtatgctatgaagcagcgtattacagtgacagttgacagcgacagctatcagttgctcaaggcatat
atgatgtcaatatctccggtctggtaagcacaaccatgcagaatgaagcccgtcgtctgcgtgccgaacgctggaaagcgg
aaaatcaggaagggatggctgaggtcgcccggtttattgaaatgaacggctcttttgctgacgagaacaggggctggtgaa
atgcagtttaaggtttacacctataaaagagagagccgttatcgtctgtttgtggatgtacagagtgatattattgacacgcccg
ggcgacggatggtgatccccctggccagtgcacgtctgctgtcagataaagtctcccgtgaactttacccggtggtgcatat
cggggatgaaagctggcgcatgatgaccaccgatatggccagtgtgccggtatccgttatcggggaagaagtggctgatc
tcagccaccgcgaaaatgacatcaaaaacgccattaacctgatgttctggggaatataaggtctcagttttagagctagaaat
agcaagttaaaataaggctagtccgttatcaacttgaaaaagtggcaccgagtcggtgctttttttaaagaattctcgacctcg

agacaaatggcagtattcatccacaattttaaaagaaaaggggggattggggggtacagtgcaggggaaagaatagtaga
cataatagcaacagacatacaaactaaagaattacaaaaacaaattacaaaaattcaaaattttcgggtttattacagggaca
gcagagatccactttggccgcggctcgagggggttggggttgcgccttttccaaggcagccctgggtttgcgcagggacg
cggctgctctgggcgtggttccgggaaacgcagcggcgccgaccctgggactcgcacattcttcacgtccgttcgcagcg
tcacccggatcttcgccgctacccttgtgggccccccggcgacgcttcctgctccgcccctaagtcgggaaggttccttgc
ggttcgcggcgtgccggacgtgacaaacggaagccgcacgtctcactagtaccctcgcagacggacagcgccaggga
gcaatggcagcgcgccgaccgcgatgggctgtggccaatagcggctgctcagcagggcgcgccgagagcagcggcc
gggaaggggcggtgcgggaggcggggtgtggggcggtagtgtgggccctgttcctgcccgcgcggtgttccgcattct
gcaagcctccggagcgcacgtcggcagtcggctccctcgttgaccgaatcaccgacctctctccccagggggatccacc
ggagcttaccatgaccgagtacaagcccacggtgcgcctcgccacccgcgacgacgtccccagggccgtacgcaccct
cgccgccgcgttcgccgactaccccgccacgcgccacaccgtcgatccggaccgccacatcgagcgggtcaccgagct
gcaagaactcttcctcacgcgcgtcgggctcgacatcggcaaggtgtgggtcgcggacgacggcgccgcggtggcggt
ctggaccacgccggagagcgtcgaagcgggggcggtgttcgccgagatcggcccgcgcatggccgagttgagcggtt
cccggctggccgcgcagcaacagatggaaggcctcctggcgccgcaccggcccaaggagcccgcgtggttcctggcc
accgtcggcgtctcgcccgaccaccagggcaagggtctgggcagcgccgtcgtgctccccggagtggaggcggccga
gcgcgccggggtgcccgccttcctggaaacctccgcgccccgcaacctccccttctacgagcggctcggcttcaccgtca
ccgccgacgtcgaggtgcccgaaggaccgcgcacctggtgcatgacccgcaagcccggtgcctgacgcccgccccac
gacccgcagcgcccgaccgaaaggagcgcacgaccccatgcatcggtacctttaagaccaatgacttacaaggcagctg
tagatcttagccactttctagagtcggggcggccggccgcttcgagcagacatgataagatacattgatgagtttggacaaa
ccacaactagaatgcagtgaaaaaaatgctttatttgtgaaatttgtgatgctattgctttatttgtaaccattataagctgcaata
aacaagttaacaacaacaattgcattcattttatgtttcaggttcagggggaggtgtgggaggttttttaaagcaagtaaaacct
ctacaaatgtggtaaaatcgataaggatccgtcgaccgatgcccttgagagccttcaacccagtcagctccttccggtgggc
gcggggcatgactatcgtcgccgcacttatgactgtcttctttatcatgcaactcgtaggacaggtgccggcagcgctcttcc
gcttcctcgctcactgactcgctgcgctcggtcgttcggctgcggcgagcggtatcagctcactcaaaggcggtaatacgg
ttatccacagaatcaggggataacgcaggaaagaacatgtgagcaaaaggccagcaaaaggccaggaaccgtaaaaag
gccgcgttgctggcgtttttccataggctccgcccccctgacgagcatcacaaaaatcgacgctcaagtcagaggtggcga
aacccgacaggactataaagataccaggcgtttccccctggaagctccctcgtgcgctctcctgttccgaccctgccgctta
ccggatacctgtccgcctttctcccttcgggaagcgtggcgctttctcaatgctcacgctgtaggtatctcagttcggtgtagg
tcgttcgctccaagctgggctgtgtgcacgaaccccccgttcagcccgaccgctgcgccttatccggtaactatcgtcttga
gtccaacccggtaagacacgacttatcgccactggcagcagccactggtaacaggattagcagagcgaggtatgtaggc
ggtgctacagagttcttgaagtggtggcctaactacggctacactagaaggacagtatttggtatctgcgctctgctgaagcc
agttaccttcggaaaaagagttggtagctcttgatccggcaaacaaaccaccgctggtagcggtggtttttttgtttgcaagca
gcagattacgcgcagaaaaaaaggatctcaagaagatcctttgatcttttctacggggtctgacgctcagtggaacgaaaac
tcacgttaagggattttggtcatgagattatcaaaaaggatcttcacctagatccttttaaattaaaaatgaagttttaaatcaatc
taaagtatatatgagtaaacttggtctgacagttaccaatgcttaatcagtgaggcacctatctcagcgatctgtctatttcgttc
atccatagttgcctgactccccgtcgtgtagataactacgatacgggagggcttaccatctggccccagtgctgcaatgata
ccgcgggacccacgctcaccggctccagatttatcagcaataaaccagccagccggaagggccgagcgcagaagtggt
cctgcaactttatccgcctccatccagtctattaattgttgccgggaagctagagtaagtagttcgccagttaatagtttgcgca
acgttgttgccattgctacaggcatcgtggtgtcacgctcgtcgtttggtatggcttcattcagctccggttcccaacgatcaa
ggcgagttacatgatcccccatgttgtgcaaaaaagcggttagctccttcggtcctccgatcgttgtcagaagtaagttggcc
gcagtgttatcactcatggttatggcagcactgcataattctcttactgtcatgccatccgtaagatgcttttctgtgactggtga
gtactcaaccaagtcattctgagaatagtgtatgcggcgaccgagttgctcttgcccggcgtcaatacgggataataccgcg
ccacatagcagaactttaaaagtgctcatcattggaaaacgttcttcggggcgaaaactctcaaggatcttaccgctgttgag
atccagttcgatgtaacccactcgtgcacccaactgatcttcagcatcttttactttcaccagcgtttctgggtgagcaaaaaca

ggaaggcaaaatgccgcaaaaaagggaataagggcgacacggaaatgttgaatactcatactcttcctttttcaatattattg
aagcatttatcagggttattgtctcatgagcggatacatatttgaatgtatttagaaaaataaacaaataggggttccgcgcaca
tttccccgaaaagtgccacctgacgcgccctgtagcggcgcattaagcgcggcgggtgtggtggttacgcgcagcgtgac
cgctacacttgccagcgccctagcgcccgctcctttcgctttcttcccttcctttctcgccacgttcgccggctttccccgtcaa
gctctaaatcgggggctccctttagggttccgatttagtgctttacggcacctcgaccccaaaaaacttgattagggtgatggt
tcacgtagtgggccatcgccctgatagacggtttttcgccctttgacgttggagtccacgttctttaatagtggactcttgttcca
aactggaacaacactcaaccctatctcggtctattcttttgatttataagggattttgccgatttcggcctattggttaaaaaatga
gctgatttaacaaaaatttaacgcgaattttaacaaaatattaacgtttacaatttcccattcgccattcaggctgcgcaactgtt
gggaagggcgatcggtgcgggcctcttcgctattacgccagcccaagctaccatgataagtaagtaatattaaggtacggg
aggtacttggagcggccgcaataaaatatctttattttcattacatctgtgtgttggttttttgtgtgaatcgatagtactaacatac
gctctccatcaaaacaaaacgaaacaaaacaaactagcaaaataggctgtccccagtgcaagtgcaggtgccagaacattt
ctctatcgata

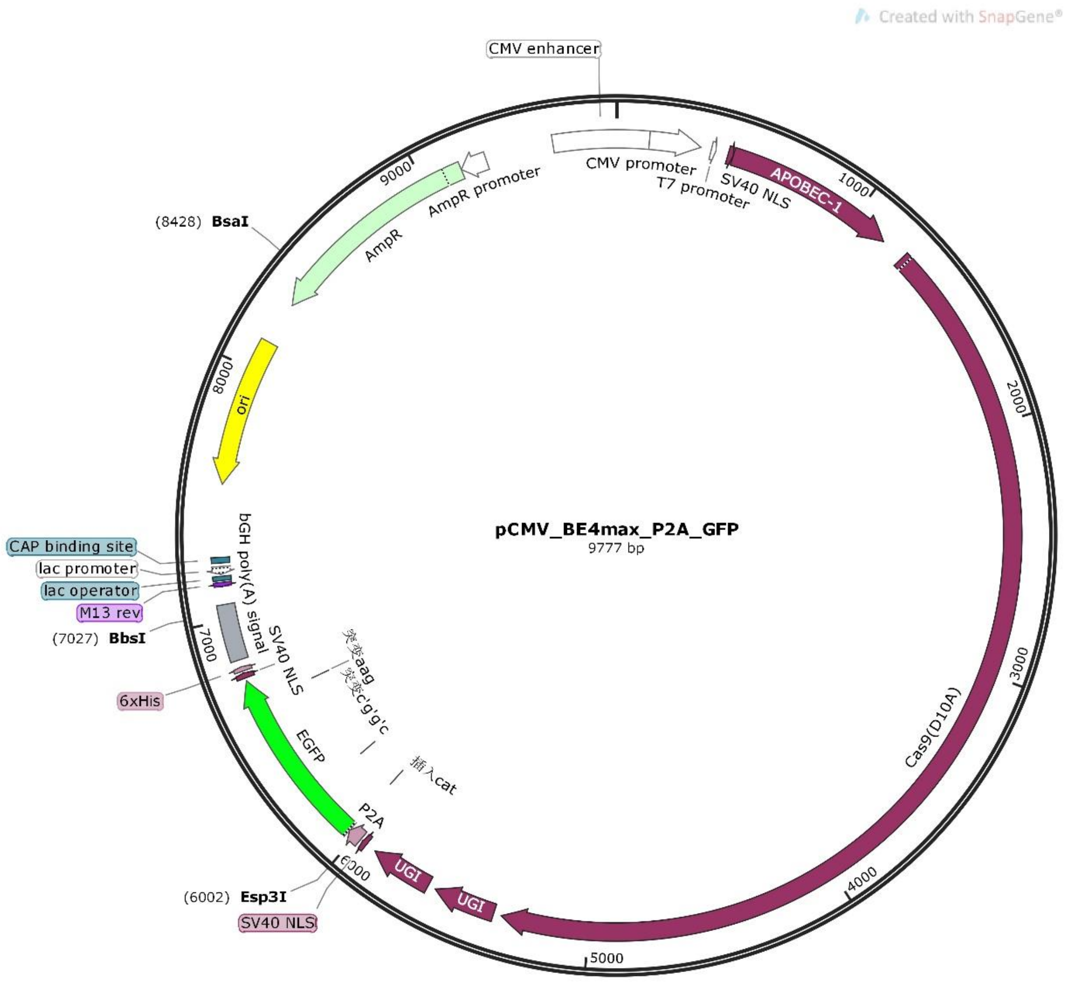

Supplementary Fig. 9 BE4max plasmid map

BE4max plasmid sequence:

atatgccaagtacgccccctattgacgtcaatgacggtaaatggcccgcctggcattatgcccagtacatgaccttatggga
ctttcctacttggcagtacatctacgtattagtcatcgctattaccatggtgatgcggttttggcagtacatcaatgggcgtggat
agcggtttgactcacggggatttccaagtctccaccccattgacgtcaatgggagtttgttttggcaccaaaatcaacgggac
tttccaaaatgtcgtaacaactccgccccattgacgcaaatgggcggtaggcgtgtacggtgggaggtctatataagcaga
gctggtttagtgaaccgtcagatccgctagagatccgcggccgctaatacgactcactatagggagagccgccaccatga
aacggacagccgacggaagcgagttcgagtcaccaaagaagaagcggaaagtctcctcagagactgggcctgtcgccg
tcgatccaaccctgcgccgccggattgaacctcacgagtttgaagtgttctttgacccccgggagctgagaaaggagacat
gcctgctgtacgagatcaactggggaggcaggcactccatctggaggcacacctctcagaacacaaataagcacgtgga
ggtgaacttcatcgagaagtttaccacagagcggtacttctgccccaataccagatgtagcatcacatggtttctgagctggt
ccccttgcggagagtgtagcagggccatcaccgagttcctgtccagatatccacacgtgacactgtttatctacatcgccag
gctgtatcaccacgcagacccaaggaataggcagggcctgcgcgatctgatcagctccggcgtgaccatccagatcatga
cagagcaggagtccggctactgctggcggaacttcgtgaattattctcctagcaacgaggcccactggcctaggtacccac
acctgtgggtgcgcctgtacgtgctggagctgtattgcatcatcctgggcctgccccccttgtctgaatatcctgcggagaaag
cagccccagctgaccttctttacaatcgccctgcagtcttgtcactatcagaggctgccaccccacatcctgtgggccacag
gcctgaagtctggaggatctagcggaggatcctctggcagcgagacaccaggaacaagcgagtcagcaacaccagaga
gcagtggcggcagcagcggcggcagcgacaagaagtacagcatcggcctggccatcggcaccaactctgtgggctgg
gccgtgatcaccgacgagtacaaggtgcccagcaagaaattcaaggtgctgggcaacaccgaccggcacagcatcaag

aagaacctgatcggagccctgctgttcgacagcggcgaaacagccgaggccacccggctgaagagaaccgccagaag
aagatacaccagacggaagaaccggatctgctatctgcaagagatcttcagcaacgagatggccaaggtggacgacagc
ttcttccacagactggaagagtccttcctggtggaagaggataagaagcacgagcggcaccccatcttcggcaacatcgtg
gacgaggtggcctaccacgagaagtaccccaccatctaccacctgagaaagaaactggtggacagcaccgacaaggcc
gacctgcggctgatctatctggccctggcccacatgatcaagttccggggccacttcctgatcgagggcgacctgaacccc
gacaacagcgacgtggacaagctgttcatccagctggtgcagacctacaaccagctgttcgaggaaaaccccatcaacgc
cagcggcgtggacgccaaggccatcctgtctgccagactgagcaagagcagacggctggaaaatctgatcgcccagct
gcccggcgagaagaagaatggcctgttcggaaacctgattgccctgagcctgggcctgacccccaacttcaagagcaact
tcgacctggccgaggatgccaaactgcagctgagcaaggacacctacgacgacgacctggacaacctgctggcccaga
tcggcgaccagtacgccgacctgtttctggccgccaagaacctgtccgacgccatcctgctgagcgacatcctgagagtg
aacaccgagatcaccaaggcccccctgagcgcctctatgatcaagagatacgacgagcaccaccaggacctgaccctgc
tgaaagctctcgtgcggcagcagctgcctgagaagtacaaagagattttcttcgaccagagcaagaacggctacgccggc
tacattgacggcggagccagccaggaagagttctacaagttcatcaagcccatcctggaaaagatggacggcaccgagg
aactgctcgtgaagctgaacagagaggacctgctgcggaagcagcggaccttcgacaacggcagcatcccccaccaga
tccacctgggagagctgcacgccattctgcggcggcaggaagatttttacccattcctgaaggacaaccgggaaaagatc
gagaagatcctgaccttccgcatcccctactacgtgggccctctggccaggggaaacagcagattcgcctggatgaccag
aaagagcgaggaaaccatcaccccctggaacttcgaggaagtggtggacaagggcgcttccgcccagagcttcatcgag
cggatgaccaacttcgataagaacctgcccaacgagaaggtgctgcccaagcacagcctgctgtacgagtacttcaccgt
gtataacgagctgaccaaagtgaaatacgtgaccgagggaatgagaaagcccgccttcctgagcggcgagcagaaaaa
ggccatcgtggacctgctgttcaagaccaaccggaaagtgaccgtgaagcagctgaaagaggactacttcaagaaaatcg
agtgcttcgactccgtggaaatctccggcgtggaagatcggttcaacgcctccctgggcacataccacgatctgctgaaaat
tatcaaggacaaggacttcctggacaatgaggaaaacgaggacattctggaagatatcgtgctgaccctgacactgtttga
ggacagagagatgatcgaggaacggctgaaaacctatgcccacctgttcgacgacaaagtgatgaagcagctgaagcg
gcggagatacaccggctggggcaggctgagccggaagctgatcaacggcatccgggacaagcagtccggcaagacaa
tcctggatttcctgaagtccgacggcttcgccaacagaaacttcatgcagctgatccacgacgacagcctgacctttaaaga
ggacatccagaaagcccaggtgtccggccagggcgatagcctgcacgagcacattgccaatctggccggcagccccgc
cattaagaagggcatcctgcagacagtgaaggtggtggacgagctcgtgaaagtgatgggccggcacaagcccgagaa
catcgtgatcgaaatggccagagagaaccagaccacccagaagggacagaagaacagccgcgagagaatgaagcgg
atcgaagagggcatcaaagagctgggcagccagatcctgaaagaacaccccgtggaaaacacccagctgcagaacga
gaagctgtacctgtactacctgcagaatgggcgggatatgtacgtggaccaggaactggacatcaaccggctgtccgact
acgatgtggaccatatcgtgcctcagagctttctgaaggacgactccatcgacaacaaggtgctgaccagaagcgacaag
aaccggggcaagagcgacaacgtgccctccgaagaggtcgtgaagaagatgaagaactactggcggcagctgctgaac
gccaagctgattacccagagaaagttcgacaatctgaccaaggccgagagaggcggcctgagcgaactggataaggcc
ggcttcatcaagagacagctggtggaaacccggcagatcacaaagcacgtggcacagatcctggactcccggatgaaca
ctaagtacgacgagaatgacaagctgatccgggaagtgaaagtgatcaccctgaagtccaagctggtgtccgatttccgga
aggatttccagttttacaaagtgcgcgagatcaacaactaccaccacgcccacgacgcctacctgaacgccgtcgtggga
accgccctgatcaaaaagtaccctaagctggaaagcgagttcgtgtacggcgactacaaggtgtacgacgtgcggaagat
gatcgccaagagcgagcaggaaatcggcaaggctaccgccaagtacttcttctacagcaacatcatgaactttttcaagac
cgagattaccctggccaacggcgagatccggaagcggcctctgatcgagacaaacggcgaaaccggggagatcgtgtg
ggataagggccgggattttgccaccgtgcggaaagtgctgagcatgccccaagtgaatatcgtgaaaaagaccgaggtg
cagacaggcggcttcagcaaagagtctatcctgcccaagaggaacagcgataagctgatcgccagaaagaaggactgg
gaccctaagaagtacggcggcttcgacagccccaccgtggcctattctgtgctggtggtggccaaagtggaaaagggca
agtccaagaaactgaagagtgtgaaagagctgctggggatcaccatcatggaaagaagcagcttcgagaagaatcccatc
gactttctggaagccaagggctacaaagaagtgaaaaaggacctgatcatcaagctgcctaagtactccctgttcgagctg

gaaaacggccggaagagaatgctggcctctgccggcgaactgcagaagggaaacgaactggccctgccctccaaatat
gtgaacttcctgtacctggccagccactatgagaagctgaagggctcccccgaggataatgagcagaaacagctgtttgtg
gaacagcacaagcactacctggacgagatcatcgagcagatcagcgagttctccaagagagtgatcctggccgacgcta
atctggacaaagtgctgtccgcctacaacaagcaccgggataagcccatcagagagcaggccgagaatatcatccacctg
tttaccctgaccaatctgggagcccctgccgccttcaagtactttgacaccaccatcgaccggaagaggtacaccagcacc
aaagaggtgctggacgccaccctgatccaccagagcatcaccggcctgtacgagacacggatcgacctgtctcagctgg
gaggtgacagcggcgggagcggcgggagcgggggagcactaatctgagcgacatcattgagaaggagactgggaa
acagctggtcattcaggagtccatcctgatgctgcctgaggaggtggaggaagtgatcggcaacaagccagagtctgaca
tcctggtgcacaccgcctacgacgagtccacagatgagaatgtgatgctgctgacctctgacgcccccgagtataagcctt
gggccctggtcatccaggattctaacggcgagaataagatcaagatgctgagcggaggatccggaggatctggaggcag
caccaacctgtctgacatcatcgagaaggagacaggcaagcagctggtcatccaggagagcatcctgatgctgcccgaa
gaagtcgaagaagtgatcggaaacaagcctgagagcgatatcctggtccataccgcctacgacgagagtaccgacgaaa
atgtgatgctgctgacatccgacgccccagagtataagccctgggctctggtcatccaggattccaacggagagaacaaa
atcaaaatgctgtctggcggctcaaaaagaaccgccgacggcagcgaattcgagcccaagaagaagaggaaagtcgga
agcggagctactaacttcagcctgctgaagcaggctggagacgtggaggagaaccctggacctatggtgagcaagggc
gaggagctgttcaccggggtggtgcccatcctggtcgagctggacggcgacgtaaacggccacaagttcagcgtgtccg
gcgagggcgagggcgatgccacctacggcaagctgaccctgaagttcatctgcaccaccggcaagctgcccgtgccct
ggcccaccctcgtgaccaccctgacctatggagtgcagtgcttcagccgctaccccgaccacatgaagcagcacgacttc
ttcaagtccgccatgcccgaaggctacgtccaggagcgcaccatcttcttcaaggacgacggcaactacaagacccgcgc
cgaggtgaagttcgagggcgacaccctggtgaaccgcatcgagctgaagggcatcgacttcaaggaggacggcaacat
cctggggcacaagctggagtacaactacaacagccacaacgtctatatcatggccgacaagcagaagaacggcatcaag
gtgaacttcaagatccgccacaacatcgaggacggcagcgtgcagctcgccgaccactaccagcagaacacccccatcg
gcgacggccccgtgctgctgcccgacaaccactacctgagcacccagtccgccctgagcaaagaccccaacgagaagc
gcgatcacatggtcctgctggagttcgtgaccgccgccgggatcactctcggcatggacgagctgtacaagtctggtggtt
ctcccaagaagaagaggaaagtctaaccggtcatcatcaccatcaccattgagtttaaacccgctgatcagcctcgactgtg
ccttctagttgccagccatctgttgtttgcccctcccccgtgccttccttgaccctggaaggtgccactcccactgtcctttccta
ataaaatgaggaaattgcatcgcattgtctgagtaggtgtcattctattctggggggtggggtggggcaggacagcaaggg
ggaggattgggaagacaatagcaggcatgctggggatgcggtgggctctatggcttctgaggcggaaagaaccagctgg
ggctcgataccgtcgacctctagctagagcttggcgtaatcatggtcatagctgtttcctgtgtgaaattgttatccgctcacaa
ttccacacaacatacgagccggaagcataaagtgtaaagcctagggtgcctaatgagtgagctaactcacattaattgcgtt
gcgctcactgcccgctttccagtcgggaaacctgtcgtgccagctgcattaatgaatcggccaacgcgcggggagaggc
ggtttgcgtattgggcgctcttccgcttcctcgctcactgactcgctgcgctcggtcgttcggctgcggcgagcggtatcagc
tcactcaaaggcggtaatacggttatccacagaatcaggggataacgcaggaaagaacatgtgagcaaaaggccagcaa
aaggccaggaaccgtaaaaaggccgcgttgctggcgtttttccataggctccgcccccctgacgagcatcacaaaaatcg
acgctcaagtcagaggtggcgaaacccgacaggactataaagataccaggcgtttccccctggaagctccctcgtgcgct
ctcctgttccgaccctgccgcttaccggatacctgtccgcctttctcccttcgggaagcgtggcgctttctcatagctcacgct
gtaggtatctcagttcggtgtaggtcgttcgctccaagctgggctgtgtgcacgaaccccccgttcagcccgaccgctgcg
ccttatccggtaactatcgtcttgagtccaacccggtaagacacgacttatcgccactggcagcagccactggtaacaggat
tagcagagcgaggtatgtaggcggtgctacagagttcttgaagtggtggcctaactacggctacactagaagaacagtattt
ggtatctgcgctctgctgaagccagttaccttcggaaaaagagttggtagctcttgatccggcaaacaaaccaccgctggta
gcggtggtttttttgtttgcaagcagcagattacgcgcagaaaaaaaggatctcaagaagatcctttgatcttttctacggggt
ctgacactcagtggaacgaaaactcacgttaagggattttggtcatgagattatcaaaaaggatcttcacctagatccttttaa
attaaaaatgaagttttaaatcaatctaaagtatatatgagtaaacttggtctgacagttaccaatgcttaatcagtgaggcacct
atctcagcgatctgtctatttcgttcatccatagttgcctgactccccgtcgtgtagataactacgatacgggagggcttaccat

ctggccccagtgctgcaatgataccgcgagacccacgctcaccggctccagatttatcagcaataaaccagccagccgga
agggccgagcgcagaagtggtcctgcaactttatccgcctccatccagtctattaattgttgccgggaagctagagtaagta
gttcgccagttaatagtttgcgcaacgttgttgccattgctacaggcatcgtggtgtcacgctcgtcgtttggtatggcttcattc
agctccggttcccaacgatcaaggcgagttacatgatcccccatgttgtgcaaaaaagcggttagctccttcggtcctccgat
cgttgtcagaagtaagttggccgcagtgttatcactcatggttatggcagcactgcataattctcttactgtcatgccatccgta
agatgcttttctgtgactggtgagtactcaaccaagtcattctgagaatagtgtatgcggcgaccgagttgctcttgcccggc
gtcaatacgggataataccgcgccacatagcagaactttaaaagtgctcatcattggaaaacgttcttcggggcgaaaactc
tcaaggatcttaccgctgttgagatccagttcgatgtaacccactcgtgcacccaactgatcttcagcatcttttactttcacca
gcgtttctgggtgagcaaaaacaggaaggcaaaatgccgcaaaaaagggaataagggcgacacggaaatgttgaatact
catactcttcctttttcaatattattgaagcatttatcagggttattgtctcatgagcggatacatatttgaatgtatttagaaaaata
aacaaataggggttccgcgcacatttccccgaaaagtgccacctgacgtcgacggatcgggagatcgatctcccgatccc
ctagggtcgactctcagtacaatctgctctgatgccgcatagttaagccagtatctgctccctgcttgtgtgttggaggtcgct
gagtagtgcgcgagcaaaatttaagctacaacaaggcaaggcttgaccgacaattgcatgaagaatctgcttagggttagg
cgttttgcgctgcttcgcgatgtacgggccagatatacgcgttgacattgattattgactagttattaatagtaatcaattacggg
gtcattagttcatagcccatatatggagttccgcgttacataacttacggtaaatggcccgcctggctgaccgcccaacgacc
cccgcccattgacgtcaataatgacgtatgttcccatagtaacgccaatagggactttccattgacgtcaatgggtggagtatt
tacggtaaactgcccacttggcagtacatcaagtgtatc

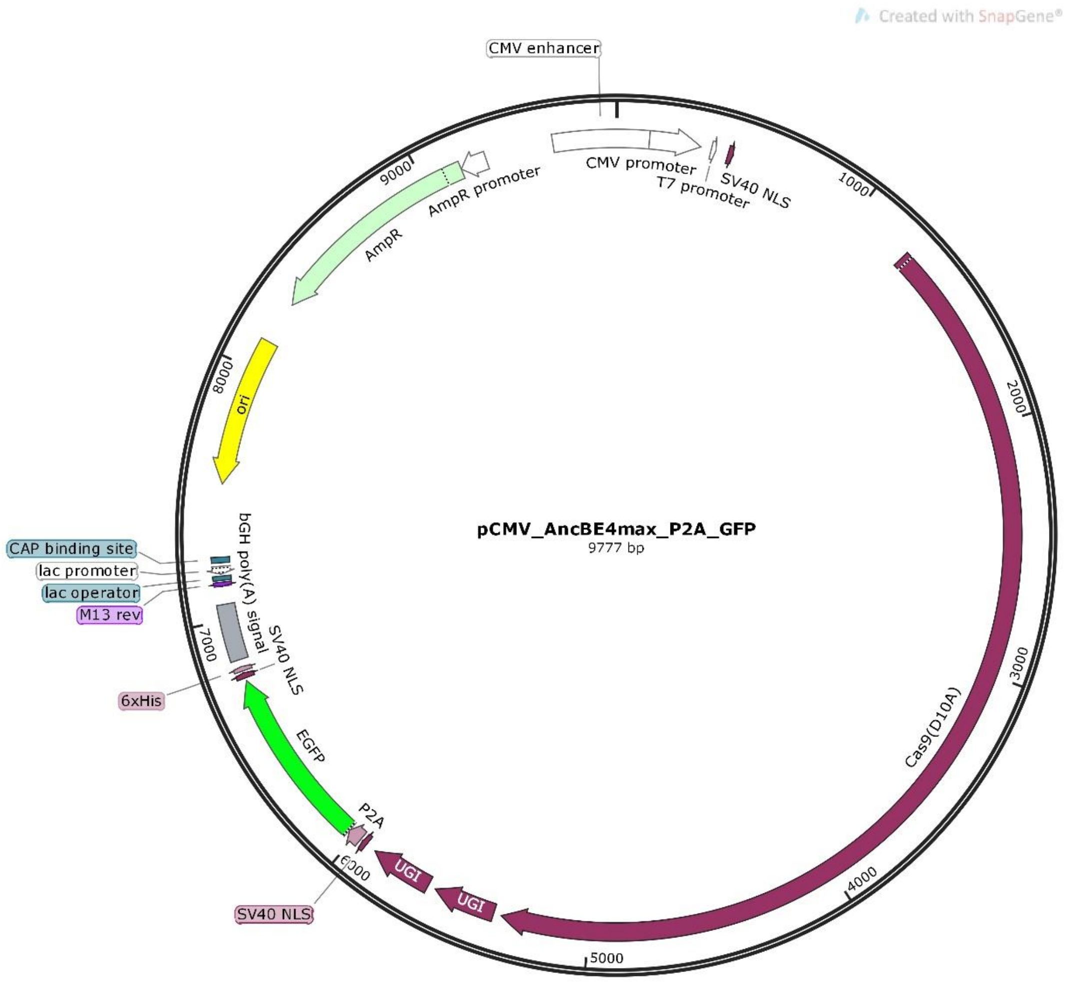

Supplementary Fig. 10 Anc-BE4max plasmid map

Anc-BE4max plasmid sequence:

atatgccaagtacgccccctattgacgtcaatgacggtaaatggcccgcctggcattatgcccagtacatgaccttatgggactttcctacttgg
cagtacatctacgtattagtcatcgctattaccatggtgatgcggttttggcagtacatcaatgggcgtggatagcggtttgactcacggggattt
ccaagtctccaccccattgacgtcaatgggagtttgttttggcaccaaaatcaacgggactttccaaaatgtcgtaacaactccgccccattgac
gcaaatgggcggtaggcgtgtacggtgggaggtctatataagcagagctggtttagtgaaccgtcagatccgctagagatccgcggccgcta
atacgactcactatagggagagccgccaccatgaaacggacagccgacggaagcgagttcgagtcaccaaagaagaagcggaaagtcag
cagtgaaaccggaccagtggcagtggacccaaccctgaggagacggattgagccccatgaatttgaagtgttctttgacccaagggagctg
aggaaggagacatgcctgctgtacgagatcaagtggggcacaagccacaagatctggcgccacagctccaagaacaccacaaagcacgt
ggaagtgaatttcatcgagaagtttacctccgagcggcacttctgcccctctaccagctgttccatcacatggtttctgtcttggagcccttgcgg
cgagtgttccaaggccatcaccgagttcctgtctcagcaccctaacgtgaccctggtcatctacgtggcccggctgtatcaccacatggacca
gcagaacaggcagggcctgcgcgatctggtgaattctggcgtgaccatccagatcatgacagccccagagtacgactattgctggcggaac
ttcgtgaattatccacctggcaaggaggcacactggccaagatacccacccctgtggatgaagctgtatgcactggagctgcacgcaggaat
cctgggcctgcctccatgtctgaatatcctgcggagaaagcagccccagctgacatttttcaccattgctctgcagtcttgtcactatcagcggct
gcctcctcatattctgtgggctacaggcctgaagtctggaggatctagcggaggatcctctggcagcgagacaccaggaacaagcgagtca
gcaacaccagagagcagtggcggcagcagcggcggcagcgacaagaagtacagcatcggcctggccatcggcaccaactctgtgggct
gggccgtgatcaccgacgagtacaaggtgcccagcaagaaattcaaggtgctgggcaacaccgaccggcacagcatcaagaagaacctg
atcggagccctgctgttcgacagcggcgaaacagccgaggccacccggctgaagagaaccgccagaagaagatacaccagacggaaga
accggatctgctatctgcaagagatcttcagcaacgagatggccaaggtggacgacagcttcttccacagactggaagagtccttcctggtgg

aagaggataagaagcacgagcggcaccccatcttcggcaacatcgtggacgaggtggcctaccacgagaagtaccccaccatctaccacc
tgagaaagaaactggtggacagcaccgacaaggccgacctgcggctgatctatctggccctggcccacatgatcaagttccggggccactt
cctgatcgagggcgacctgaaccccgacaacagcgacgtggacaagctgttcatccagctggtgcagacctacaaccagctgttcgaggaa
aaccccatcaacgccagcggcgtggacgccaaggccatcctgtctgccagactgagcaagagcagacggctggaaaatctgatcgcccag
ctgcccggcgagaagaagaatggcctgttcggaaacctgattgccctgagcctgggcctgacccccaacttcaagagcaacttcgacctgg
ccgaggatgccaaactgcagctgagcaaggacacctacgacgacgacctggacaacctgctggcccagatcggcgaccagtacgccgac
ctgtttctggccgccaagaacctgtccgacgccatcctgctgagcgacatcctgagagtgaacaccgagatcaccaaggcccccctgagcg
cctctatgatcaagagatacgacgagcaccaccaggacctgaccctgctgaaagctctcgtgcggcagcagctgcctgagaagtacaaaga
gattttcttcgaccagagcaagaacggctacgccggctacattgacggcggagccagccaggaagagttctacaagttcatcaagcccatcc
tggaaaagatggacggcaccgaggaactgctcgtgaagctgaacagagaggacctgctgcggaagcagcggaccttcgacaacggcag
catccccccaccagatccacctgggagagctgcacgccattctgcggcggcaggaagatttttacccattcctgaaggacaaccgggaaaag
atcgagaagatcctgaccttccgcatcccctactacgtgggccctctggccaggggaaacagcagattcgcctggatgaccagaaagagcg
aggaaaccatcaccccctggaacttcgaggaagtggtggacaagggcgcttccgcccagagcttcatcgagcggatgaccaacttcgataa
gaacctgcccaacgagaaggtgctgcccaagcacagcctgctgtacgagtacttcaccgtgtataacgagctgaccaaagtgaaatacgtga
ccgagggaatgagaaagcccgccttcctgagcggcgagcagaaaaaggccatcgtggacctgctgttcaagaccaaccggaaagtgacc
gtgaagcagctgaaagaggactacttcaagaaaatcgagtgcttcgactccgtggaaatctccggcgtggaagatcggttcaacgcctccct
gggcacataccacgatctgctgaaaattatcaaggacaaggacttcctggacaatgaggaaaacgaggacattctggaagatatcgtgctga
ccctgacactgtttgaggacagagagatgatcgaggaacggctgaaaacctatgcccacctgttcgacgacaaagtgatgaagcagctgaa
gcggcggagatacaccggctggggcaggctgagccggaagctgatcaacggcatccgggacaagcagtccggcaagacaatcctggatt
tcctgaagtccgacggcttcgccaacagaaacttcatgcagctgatccacgacgacagcctgacctttaaagaggacatccagaaagcccag
gtgtccggccagggcgatagcctgcacgagcacattgccaatctggccggcagccccgccattaagaagggcatcctgcagacagtgaag
gtggtggacgagctcgtgaaagtgatgggccggcacaagcccgagaacatcgtgatcgaaatggccagagagaaccagaccacccagaa
gggacagaagaacagccgcgagagaatgaagcggatcgaagagggcatcaaagagctgggcagccagatcctgaaagaacaccccgt
ggaaaacacccagctgcagaacgagaagctgtacctgtactacctgcagaatgggcgggatatgtacgtggaccaggaactggacatcaac
cggctgtccgactacgatgtggaccatatcgtgcctcagagctttctgaaggacgactccatcgacaacaaggtgctgaccagaagcgacaa
gaaccggggcaagagcgacaacgtgccctccgaagaggtcgtgaagaagatgaagaactactggcggcagctgctgaacgccaagctga
ttacccagagaaagttcgacaatctgaccaaggccgagagaggcggcctgagcgaactggataaggccggcttcatcaagagacagctgg
tggaaacccggcagatcacaaagcacgtggcacagatcctggactcccggatgaacactaagtacgacgagaatgacaagctgatccggg
aagtgaaagtgatcaccctgaagtccaagctggtgtccgatttccggaaggatttccagttttacaaagtgcgcgagatcaacaactaccacca
cgcccacgacgcctacctaaacgccgtcgtgggaaccgccctgatcaaaaagtaccctaagctggaaagcgagttcgtgtacggcgactac
aaggtgtacgacgtgcggaagatgatcgccaagagcgagcaggaaatcggcaaggctaccgccaagtacttcttctacagcaacatcatga
actttttcaagaccgagattaccctggccaacggcgagatccggaagcggcctctgatcgagacaaacggcgaaaccggggagatcgtgtg
ggataagggccgggattttgccaccgtgcggaaagtgctgagcatgccccaagtgaatatcgtgaaaaagaccgaggtgcagacaggcgg
cttcagcaaagagtctatcctgcccaagaggaacagcgataagctgatcgccagaaagaaggactgggaccctaagaagtacggcggcttc
gacagccccaccgtggcctattctgtgctggtggtggccaaagtggaaaagggcaagtccaagaaactgaagagtgtgaaagagctgctgg
ggatcaccatcatggaaagaagcagcttcgagaagaatcccatcgactttctggaagccaagggctacaaagaagtgaaaaaggacctgat
catcaagctgcctaagtactccctgttcgagctggaaaacggccggaagagaatgctggcctctgccggcgaactgcagaagggaaacga
actggccctgccctccaaatatgtgaacttcctgtacctggccagccactatgagaagctgaagggctcccccgaggataatgagcagaaac
agctgtttgtggaacagcacaagcactacctggacgagatcatcgagcagatcagcgagttctccaagagagtgatcctggccgacgctaat
ctggacaaagtgctgtccgcctacaacaagcaccgggataagcccatcagagagcaggccgagaatatcatccacctgtttaccctgaccaa
tctgggagcccctgccgccttcaagtactttgacaccaccatcgaccggaagaggtacaccagcaccaaagaggtgctggacgccaccctg
atccaccagagcatcaccggcctgtacgagacacggatcgacctgtctcagctgggaggtgacagcggcgggagcggcgggagcgggg
ggagcactaatctgagcgacatcattgagaaggagactgggaaacagctggtcattcaggagtccatcctgatgctgcctgaggaggtgga
ggaagtgatcggcaacaagccagagtctgacatcctggtgcacaccgcctacgacgagtccacagatgagaatgtgatgctgctgacctctg

acgcccccgagtataagccttgggccctggtcatccaggattctaacggcgagaataagatcaagatgctgagcggaggatccggaggatc
tggaggcagcaccaacctgtctgacatcatcgagaaggagacaggcaagcagctggtcatccaggagagcatcctgatgctgcccgaaga
agtcgaagaagtgatcggaaacaagcctgagagcgatatcctggtccataccgcctacgacgagagtaccgacgaaaatgtgatgctgctg
acatccgacgccccagagtataagccctgggctctggtcatccaggattccaacggagagaacaaaatcaaaatgctgtctggcggctcaaa
aagaaccgccgacggcagcgaattcgagcccaagaagaagaggaaagtcggaagcggagctactaacttcagcctgctgaagcaggctg
gagacgtggaggagaaccctggacctatggtgagcaagggcgaggagctgttcaccggggtggtgcccatcctggtcgagctggacggc
gacgtaaacggccacaagttcagcgtgtccggcgagggcgagggcgatgccacctacggcaagctgaccctgaagttcatctgcaccacc
ggcaagctgcccgtgccctggcccaccctcgtgaccaccctgacctatggagtgcagtgcttcagccgctaccccgaccacatgaagcagc
acgacttcttcaagtccgccatgcccgaaggctacgtccaggagcgcaccatcttcttcaaggacgacggcaactacaagacccgcgccga
ggtgaagttcgagggcgacaccctggtgaaccgcatcgagctgaagggcatcgacttcaaggaggacggcaacatcctggggcacaagct
ggagtacaactacaacagccacaacgtctatatcatggccgacaagcagaagaacggcatcaaggtgaacttcaagatccgccacaacatc
gaggacggcagcgtgcagctcgccgaccactaccagcagaacacccccatcggcgacggccccgtgctgctgcccgacaaccactacct
gagcacccagtccgccctgagcaaagaccccaacgagaagcgcgatcacatggtcctgctggagttcgtgaccgccgccgggatcactct
cggcatggacgagctgtacaagtctggtggttctcccaagaagaaaaggaaagtctaaccggtcatcatcaccatcaccattgagtttaaacc
cgctgatcagcctcgactgtgccttctagttgccagccatctgttgtttgcccctcccccgtgccttccttgaccctggaaggtgccactcccact
gtcctttcctaataaaatgaggaaattgcatcgcattgtctgagtaggtgtcattctattctggggggtggggtggggcaggacagcaagggg
aggattgggaagacaatagcaggcatgctggggatgcggtgggctctatggcttctgaggcggaaagaaccagctggggctcgataccgtc
gacctctagctagagcttggcgtaatcatggtcatagctgtttcctgtgtgaaattgttatccgctcacaattccacacaacatacgagccggaag
cataaagtgtaaagcctaggatgcctaatgagtgagctaactcacattaattgcgttgcgctcactgcccgctttccagtcgggaaacctgtcgt
gccagctgcattaatgaatcggccaacgcgcggggaagaggcggtttgcgtattgggcgctcttccgcttcctcgctcactgactcgctgcgct
cggtcgttcggctgcggcgagcggtatcagctcactcaaaggcggtaatacggttatccacagaatcaggggataacgcaggaaagaacat
gtgagcaaaaggccagcaaaaggccaggaaccgtaaaaaggccgcgttgctggcgtttttccataggctccgcccccctgacgagcatcac
aaaaatcgacgctcaagtcagaggtggcgaaacccgacaggactataaagataccaggcgtttccccctggaagctccctcgtgcgctctcc
tgttccgaccctgccgcttaccggatacctgtccgcctttctcccttcgggaagcgtggcgctttctcatagctcacgctgtaggtatctcagttcg
gtgtaggtcgttcgctccaagctgggctgtgtgcacgaaccccccgttcagcccgaccgctgcgccttatccggtaactatcgtcttgagtcca
acccggtaagacacgacttatcgccactggcagcagccactggtaacaggattagcagagcgaggtatgtaggcggtgctacagagttcttg
aagtggtggcctaactacggctacactagaagaacagtatttggtatctgcgctctgctgaagccagttaccttcggaaaaagagttggtagct
cttgatccggcaaacaaaccaccgctggtagcggtggtttttttgtttgcaagcagcagattacgcgcagaaaaaaaggatctcaagaagatcc
tttgatcttttctacggggtctgacactcagtggaacgaaaactcacgttaagggattttggtcatgagattatcaaaaaggatcttcacctagatc
cttttaaattaaaaatgaagttttaaatcaatctaaagtatatatgagtaaacttggtctgacagttaccaatgcttaatcagtgaggcacctatctca
gcgatctgtctatttcgttcatccatagttgcctgactccccgtcgtgtagataactacgatacgggagggcttaccatctggccccagtgctgca
atgataccgcgagacccacgctcaccggctccagatttatcagcaataaaccagccagccggaagggccgagcgcagaagtggtcctgca
actttatccgcctccatccagtctattaattgttgccgggaagctagagtaagtagttcgccagttaatagtttgcgcaacgttgttgccattgctac
aggcatcgtggtgtcacgctcgtcgtttggtatggcttcattcagctccggttcccaacgatcaaggcgagttacatgatcccccatgttgtgcaa
aaaagcggttagctccttcggtcctccgatcgttgtcagaagtaagttggccgcagtgttatcactcatggttatggcagcactgcataattctctt
actgtcatgccatccgtaagatgcttttctgtgactggtgagtactcaaccaagtcattctgagaatagtgtatgcggcgaccgagttgctcttgc
ccggcgtcaatacgggataataccgcgccacatagcagaactttaaaagtgctcatcattggaaaacgttcttcggggcgaaaactctcaagg
atcttaccgctgttgagatccagttcgatgtaacccactcgtgcacccaactgatcttcagcatcttttactttcaccagcgtttctgggtgagcaaa
aacaggaaggcaaaatgccgcaaaaaagggaataagggcgacacggaaatgttgaatactcatactcttcctttttcaatattattgaagcattt
atcagggttattgtctcatgagcggatacatatttgaatgtatttagaaaaataaacaaataggggttccgcgcacatttccccgaaaagtgccac
ctgacgtcgacggatcgggagatcgatctcccgatcccctagggtcgactctcagtacaatctgctctgatgccgcatagttaagccagtatct
gctccctgcttgtgtgttggaggtcgctgagtagtgcgcgagcaaaatttaagctacaacaaggcaaggcttgaccgacaattgcatgaagaa
tctgcttagggttaggcgttttgcgctgcttcgcgatgtacgggccagatatacgcgttgacattgattattgactagttattaatagtaatcaattac
ggggtcattagttcatagcccatatatggagttccgcgttacataacttacggtaaatggcccgcctggctgaccgcccaacgacccccgccc

attgacgtcaataatgacgtatgttcccatagtaacgccaatagggactttccattgacgtcaatgggtggagtatttacggtaaactgcccactt
ggcagtacatcaagtgtatc

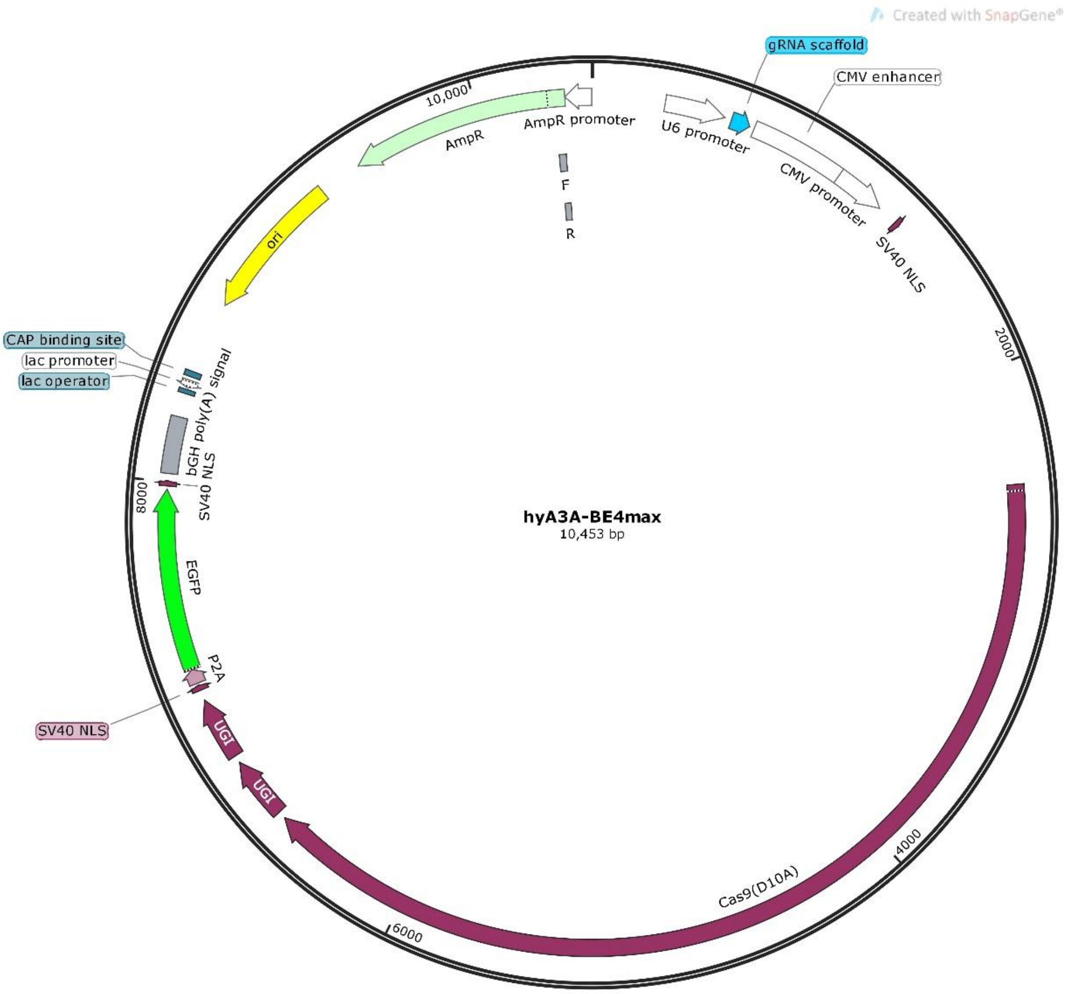

Supplementary Fig. 11 hyA3A-BE4max plasmid map

hyA3A-BE4max plasmid sequence:

cacatttccccgaaaagtgccacctgacgtcgacggatcgggagatcgatctcccgatcccctagggtcgactctcagtacaatctgctctgat
gccgcatagttaagccagtatctgctccctgcttgtgtgttggaggtcgctgagtagtgcgcgagcaaaatttaagctacaacaaggcaaggct
tgaccgacaattgcatgaagaatctgcttagggttaggcgttttgcgctgcttcgcgatgtacgggccagatatacgcgtggatccctgcagtta
attaaagagggcctatttcccatgattccttcatatttgcatatacgatacaaggctgttagagagataattagaattaatttgactgtaaacacaaa
gatattagtacaaaatacgtgacgtagaaagtaataatttcttgggtagtttgcagttttaaaattatgttttaaaatggactatcatatgcttaccgta
acttgaaagtatttcgatttcttggctttatatatcttgtggaaaggacgaaacaccgggtcttcgagaagacctgttttagagctagaaatagcaa
gttaaaataaggctagtccgttatcaacttgaaaaagtggcaccgagtcggtgctttttttatgcatggtaccgacattgattattgactagttattaa
tagtaatcaattacggggtcattagttcatagcccatatatggagttccgcgttacataacttacggtaaatggcccgcctggctgaccgcccaa
cgacccccgcccattgacgtcaataatgacgtatgttcccatagtaacgccaatagggactttccattgacgtcaatgggtggagtatttacggt
aaactgcccacttggcagtacatcaagtgtatcatatgccaagtacgccccctattgacgtcaatgacggtaaatggcccgcctggcattatgc
ccagtacatgaccttatgggactttcctacttggcagtacatctacgtattagtcatcgctattaccatggtgatgcggttttggcagtacatcaatg
ggcgtggatagcggtttgactcacggggatttccaagtctccaccccattgacgtcaatgggagtttgttttggcaccaaaatcaacgggacttt
ccaaaatgtcgtaacaactccgccccattgacgcaaatgggcggtaggcgtgtacggtgggaggtctatataagcagagctggtttagtgaac
cgtcagatccgctagagatccgcggccgccaccatgaagaggaccgccgatggctctgagttcgagagccccaagaagaagcggaaggt
ggaggcatctccagcaagcggaccaaggcacctgatggacccccacatcttcacctctaactttaacaatggcatcggcaggcacaagacat
acctgtgctatgaggtggagcgcctggacaacggcaccagcgtgaagatggatcagcacagaggcttcctgcacaaccaggccaagaatc
tgctgtgcggcttctacggccggcacgcagagctgagatttctggacctggtgcctagcctgcagctggatccagcccagatctatagggtga

cctggttcatcagctggtccccatgcttttcctggggatgtgcaggagaggtgcgcgccttcctgcaggagaatacacacgtgcggctgagaa
tctttgccgcccggatctacgactatgatcctctgtacaaggaggccctgcagatgctgagagacgcaggagcccaggtgtccatcatgacct
atgatgagttcaagcactgctgggacacatttgtggatcaccagggctgtccctttcagccttgggacggactggatgagcactcccaggccc
tgtctggcaggctgagggccatcctgcagaaccagggcaatagcggaggatctagcggaggatcaagcggaagcgagactcctggaacc
agcgaaagcgcaaccccagaaagcagcggaggaagtagcggaggaagcatggcaatgcagatgcagcttgaagcaaatgcagatacttc
agtggaagaagaaagctttggcccacaacccatttcacggttagagcagtgtggcataaatgccaacgatgtgaagaaattggaagaagctg
gattccatactgtggaggctgttgcctatgcgccaaagaaggagctaataaatattaagggaattagtgaagccaaagctgataaaattctggct
gaggcagctaaattagttccaatgggtttcaccactgcaactgaattccaccaaaggcggtcagagatcatacagattactactggctccaaag
agcttgacaaactacttcaatctggcggatctagcggtggatctagcggctctgagacccctggaacatccgaatccgccactccagagagc
agcggaggctcttctggaggatcagacaagaagtacagcatcggcctggccatcggcaccaactctgtgggctgggccgtgatcaccgac
gagtacaaggtgcccagcaagaaattcaaggtgctgggcaacaccgaccggcacagcatcaagaagaacctgatcggagccctgctgttc
gacagcggcgaaacagccgaggccacccggctgaagagaaccgccagaagaagatacaccagacggaagaaccggatctgctatctgc
aagagatcttcagcaacgagatggccaaggtggacgacagcttcttccacagactggaagagtccttcctggtggaagaggataagaagca
cgagcggcaccccatcttcggcaacatcgtggacgaggtggcctaccacgagaagtaccccaccatctaccacctgagaaagaaactggtg
gacagcaccgacaaggccgacctgcggctgatctatctggccctggcccacatgatcaagttccggggccacttcctgatcgagggcgacc
tgaaccccgacaacagcgacgtggacaagctgttcatccagctggtgcagacctacaaccagctgttcgaggaaaaccccatcaacgccag
cggcgtggacgccaaggccatcctgtctgccagactgagcaagagcagacggctggaaaatctgatcgcccagctgcccggcgagaaga
agaatggcctgttcggaaacctgattgccctgagcctgggcctgacccccaacttcaagagcaacttcgacctggccgaggatgccaaactg
cagctgagcaaggacacctacgacgacgacctggacaacctgctggcccagatcggcgaccagtacgccgacctgtttctggccgccaag
aacctgtccgacgccatcctgctgagcgacatcctgagagtgaacaccgagatcaccaaggcccccctgagcgcctctatgatcaagagata
cgacgagcaccaccaggacctgaccctgctgaaagctctcgtgcggcagcagctgcctgagaagtacaaagagattttcttcgaccagagc
aagaacggctacgccggctacattgacggcggagccagccaggaagagttctacaagttcatcaagcccatcctggaaaagatggacggc
accgaggaactgctcgtgaagctgaacagagaggacctgctgcggaagcagcggaccttcgacaacggcagcatccccccaccagatcca
cctgggagagctgcacgccattctgcggcggcaggaagatttttacccattcctgaaggacaaccgggaaaagatcgagaagatcctgacct
tccgcatcccctactacgtgggccctctggccaggggaaacagcagattcgcctggatgaccagaaagagcgaggaaaccatcacccccct
ggaacttcgaggaagtggtggacaagggcgcttccgcccagagcttcatcgagcggatgaccaacttcgataagaacctgcccaacgaga
aggtgctgcccaagcacagcctgctgtacgagtacttcaccgtgtataacgagctgaccaaagtgaaatacgtgaccgagggaatgagaaa
gcccgccttcctgagcggcgagcagaaaaaggccatcgtggacctgctgttcaagaccaaccggaaagtgaccgtgaagcagctgaaaga
ggactacttcaagaaaatcgagtgcttcgactccgtggaaatctccggcgtggaagatcggttcaacgcctccctgggcacataccacgatct
gctgaaaattatcaaggacaaggacttcctggacaatgaggaaaacgaggacattctggaagatatcgtgctgaccctgacactgtttgagga
cagagagatgatcgaggaacggctgaaaacctatgcccacctgttcgacgacaaagtgatgaagcagctgaagcggcggagatacaccgg
ctggggcaggctgagccggaagctgatcaacggcatccgggacaagcagtccggcaagacaatcctggatttcctgaagtccgacggcttc
gccaacagaaacttcatgcagctgatccacgacgacagcctgacctttaaagaggacatccagaaagcccaggtgtccggccagggcgata
gcctgcacgagcacattgccaatctggccggcagccccgccattaagaagggcatcctgcagacagtgaaggtggtggacgagctcgtga
aagtgatgggccggcacaagcccgagaacatcgtgatcgaaatggccagagagaaccagaccacccagaagggacagaagaacagcc
gcgagagaatgaagcggatcgaagagggcatcaaagagctgggcagccagatcctgaaagaacaccccgtggaaaacacccagctgca
gaacgagaagctgtacctgtactacctgcagaatgggcgggatatgtacgtggaccaggaactggacatcaaccggctgtccgactacgat
gtggaccatatcgtgcctcagagctttctgaaggacgactccatcgacaacaaggtgctgaccagaagcgacaagaaccggggcaagagc
gacaacgtgccctccgaagaggtcgtgaagaagatgaagaactactggcggcagctgctgaacgccaagctgattacccagagaaagttc
gacaatctgaccaaggccgagagaggcggcctgagcgaactggataaggccggcttcatcaagagacagctggtggaaacccggcagat
cacaaagcacgtggcacagatcctggactcccggatgaacactaagtacgacgagaatgacaagctgatccgggaagtgaaagtgatcac
cctgaagtccaagctggtgtccgatttccggaaggatttccagttttacaaagtgcgcgagatcaacaactaccaccacgcccacgacgccta
cctgaacgccgtcgtgggaaccgccctgatcaaaaagtaccctaagctggaaagcgagttcgtgtacggcgactacaaggtgtacgacgtg
cggaagatgatcgccaagagcgagcaggaaatcggcaaggctaccgccaagtacttcttctacagcaacatcatgaacttttcaagaccga

gattaccctggccaacggcgagatccggaagcggcctctgatcgagacaaacggcgaaaccggggagatcgtgtgggataagggccgg
gattttgccaccgtgcggaaagtgctgagcatgccccaagtgaatatcgtgaaaaagaccgaggtgcagacaggcggcttcagcaaagagt
ctatcctgcccaagaggaacagcgataagctgatcgccagaaagaaggactgggaccctaagaagtacggcggcttcgacagccccaccg
tggcctattctgtgctggtggtggccaaagtggaaaagggcaagtccaagaaactgaagagtgtgaaagagctgctggggatcaccatcatg
gaaagaagcagcttcgagaagaatcccatcgactttctggaagccaagggctacaaagaagtgaaaaaggacctgatcatcaagctgccta
agtactccctgttcgagctggaaaacggccggaagagaatgctggcctctgccggcgaactgcagaagggaaacgaactggccctgccct
ccaaatatgtgaacttcctgtacctggccagccactatgagaagctgaagggctcccccgaggataatgagcagaaacagctgtttgtggaac
agcacaagcactacctggacgagatcatcgagcagatcagcgagttctccaagagagtgatcctggccgacgctaatctggacaaagtgct
gtccgcctacaacaagcaccgggataagcccatcagagagcaggccgagaatatcatccacctgtttaccctgaccaatctgggagcccctg
ccgccttcaagtactttgacaccaccatcgaccggaagaggtacaccagcaccaaagaggtgctggacgccaccctgatccaccagagcat
caccggcctgtacgagacacggatcgacctgtctcagctgggaggtgacagcggcgggagcggcgggagcgggggggagcactaatctg
agcgacatcattgagaaggagactgggaaacagctggtcattcaggagtccatcctgatgctgcctgaggaggtggaggaagtgatcggca
acaagccagagtctgacatcctggtgcacaccgcctacgacgagtccacagatgagaatgtgatgctgctgacctctgacgcccccgagtat
aagccttgggccctggtcatccaggattctaacggcgagaataagatcaagatgctgagcggaggatccggaggatctggaggcagcacc
aacctgtctgacatcatcgagaaggagacaggcaagcagctggtcatccaggagagcatcctgatgctgcccgaagaagtcgaagaagtg
atcggaaacaagcctgagagcgatatcctggtccataccgcctacgacgagagtaccgacgaaaatgtgatgctgctgacatccgacgccc
cagagtataagccctgggctctggtcatccaggattccaacggagagaacaaaatcaaaatgctgtctggcggctcaaaaagaaccgccga
cggcagcgaattcgagcccaagaagaagaggaaagtcggaagcggagctactaacttcagcctgctgaagcaggctggagacgtggagg
agaaccctggacctatggtgagcaagggcgaggagctgttcaccggggtggtgcccatcctggtcgagctggacggcgacgtaaacggcc
acaagttcagcgtgtccggcgagggcgagggcgatgccacctacggcaagctgaccctgaagttcatctgcaccaccggcaagctgcccg
tgccctggcccaccctcgtgaccaccctgacctatggagtgcagtgcttcagccgctaccccgaccacatgaagcagcacgacttcttcaagt
ccgccatgcccgaaggctacgtccaggagcgcaccatcttcttcaaggacgacggcaactacaagacccgcgccgaggtgaagttcgagg
gcgacaccctggtgaaccgcatcgagctgaagggcatcgacttcaaggaggacggcaacatcctggggcacaagctggagtacaactaca
acagccacaacgtctatatcatggccgacaagcagaagaacggcatcaaggtgaacttcaagatccgccacaacatcgaggacggcagcg
tgcagctcgccgaccactaccagcagaacacccccatcggcgacggccccgtgctgctgcccgacaaccactacctgagcacccagtccg
ccctgagcaaagaccccaacgagaagcgcgatcacatggtcctgctggagttcgtgaccgccgccgggatcactctcggcatggacgagc
tgtacaagtctggtggttctcccaagaagaaaaggaaagtctaagtttaaacccgctgatcagcctcgactgtgccttctagttgccagccatct
gttgtttgcccctcccccgtgccttccttgaccctggaaggtgccactcccactgtcctttcctaataaaatgaggaaattgcatcgcattgtctga
gtaggtgtcattctattctggggggtggggtggggcaggacagcaagggggaggattgggaagagaatagcaggcatgctggggatgcg
gtgggctctatggcttctgaggcggaaagaaccagctggggctcgataccgtcgacctctagctagagcttggcgtaatcatggtcatagatg
tttcctgtgtgaaattgttatccgctcacaattccacacaacatacgagccggaagcataaagtgtaaagcctagggtgcctaatgagtgagcta
actcacattaattgcgttgcgctcactgcccgctttccagtcgggaaacctgtcgtgccagctgcattaatgaatcggccaacgcgcggggag
aggcggtttgcgtattgggcgctcttccgcttcctcgctcactgactcgctgcgctcggtcgttcggctgcggcgagcggtatcagctcactca
aaggcggtaatacggttatccacagaatcaggggataacgcaggaaagaacatgtgagcaaaaggccagcaaaaggccaggaaccgtaa
aaaggccgcgttgctggcgtttttccataggctccgcccccctgacgagcatcacaaaaatcgacgctcaagtcagaggtggcgaaacccga
caggactataaagataccaggcgtttccccctggaagctccctcgtgcgctctcctgttccgaccctgccgcttaccggatacctgtccgccttt
ctcccttcgggaagcgtggcgctttctcatagctcacgctgtaggtatctcagttcggtgtaggtcgttcgctccaagctgggctgtgtgcacga
accccccgttcagcccgaccgctgcgccttatccggtaactatcgtcttgagtccaacccggtaagacacgacttatcgccactggcagcagc
cactggtaacaggattagcagagcgaggtatgtaggcggtgctacagagttcttgaagtggtggcctaactacggctacactagaagaacag
tatttggtatctgcgctctgctgaagccagttaccttcggaaaaagagttggtagctcttgatccggcaaacaaaccaccgctagtagcggtggt
ttttttgtttgcaagcagcagattacgcgcagaaaaaaaggatctcaagaagatcctttgatcttttctacggggtctgacgctcagtggaacgaa
aactcacgttaagggattttggtcatgagattatcaaaaaggatcttcacctagatccttttaaattaaaaatgaagttttaaatcaatctaaagtatat
atgagtaaacttggtctgacagttaccaatgcttaatcagtgaggcacctatctcagcgatctgtctatttcgttcatccatagttgcctgactcccc
gtcgtgtagataactacgatacgggagggcttaccatctggccccagtgctgcaatgataccgcgagacccacgctcaccggctccagattta

tcagcaataaaccagccagccggaagggccgagcgcagaagtggtcctgcaactttatccgcctccatccagtctattaattgttgccgggaa
gctagagtaagtagttcgccagttaatagtttgcgcaacgttgttgccattgctacaggcatcgtggtgtcacgctcgtcgtttggtatggcttcatt
cagctccggttcccaacgatcaaggcgagttacatgatcccccatgttgtgcaaaaaagcggttagctccttcggtcctccgatcgttgtcaga
agtaagttggccgcagtgttatcactcatggttatggcagcactgcataattctcttactgtcatgccatccgtaagatgcttttctgtgactggtga
gtactcaaccaagtcattctgagaatagtgtatgcggcgaccgagttgctcttgcccggcgtcaatacgggataataccgcgccacatagcag
aactttaaaagtgctcatcattggaaaacgttcttcggggcgaaaactctcaaggatcttaccgctgttgagatccagttcgatgtaacccactcg
tgcacccaactgatcttcagcatcttttactttcaccagcgtttctgggtgagcaaaaacaggaaggcaaaatgccgcaaaaaagggaataag
ggcgacacggaaatgttgaatactcatactcttcctttttcaatattattgaagcatttatcagggttattgtctcatgagcggatacatatttgaatgt
atttagaaaaataaacaaataggggttccgcg

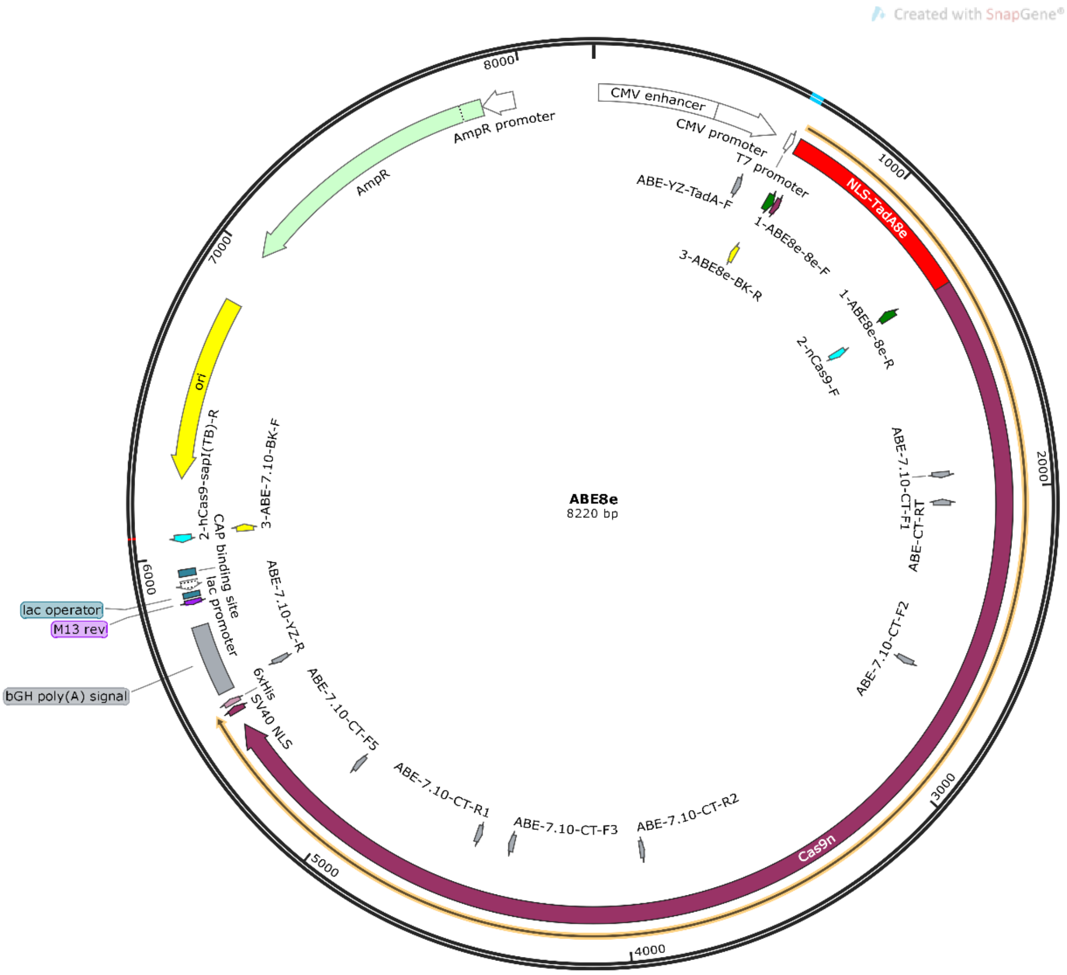

Supplementary Fig. 12 ABE8e plasmid map

ABE8e plasmid sequence:

gggccagatatacgcgttgacattgattattgactagttattaatagtaatcaattacggggtcattagttcatagcccatatatggagttccgcgtt
acataacttacggtaaatggcccgcctggctgaccgcccaacgacccccgcccattgacgtcaataatgacgtatgttcccatagtaacgcca
atagggactttccattgacgtcaatgggtggagtatttacggtaaactgcccacttggcagtacatcaagtgtatcatatgccaagtacgccccct
attgacgtcaatgacggtaaatggcccgcctggcattatgcccagtacatgaccttatgggactttcctacttggcagtacatctacgtattagtc
atcgctattaccatggtgatgcggttttggcagtacatcaatgggcgtggatagcggtttgactcacggggatttccaagtctccaccccattga
cgtcaatgggagtttgttttggcaccaaaatcaacgggactttccaaaatgtcgtaacaactccgccccattgacgcaaatgggcggtaggcgt
gtacggtgggaggtctatataagcagagctggtttagtgaaccgtcagatccgctagagatccgcggccgctaatacgactcactatagggga
gagccgccaccatgaaacggacagccgacggaagcgagttcgagtcaccaaagaagaagcggaaagtctctgaggtggagttttcccacg
agtactggatgagacatgccctgaccctggccaagagggcacgggatgagagggaggtgcctgtgggagccgtgctggtgctgaacaata
gagtgatcggcgagggctggaacagagccatcggcctgcacgacccaacagcccatgccgaaattatggccctgagacagggcggcctg
gtcatgcagaactacagactgattgacgccaccctgtacgtgacattcgagccttgcgtgatgtgcgccggcgccatgatccactctaggatc
ggccgcgtggtgtttggcgtgaggaactcaaaaagaggcgccgcaggctccctgatgaacgtgctgaactaccccggcatgaatcaccgc
gtcgaaattaccgagggaatcctggcagatgaatgtgccgccctgctgtgcgatttctatcggatgcctagacaggtgttcaatgctcagaaga
aggcccagagctccatcaactccggaggatctagcggaggctcctctggctctgagacacctggcacaagcgagagcgcaacacctgaaa
gcagcgggggcagcagcggggggtcagacaagaagtactccattgggctcgctatcggcacaaacagcgtcggctgggccgtcattacg
gacgagtacaaggtgccgagcaaaaaattcaaagttctgggcaataccgatcgccacagcataaagaagaacctcattggcgccctcctgtt
cgactccggggagacggccgaagccacgcggctcaaaagaacagcacggcgcagatatacccgcagaaagaatcggatctgctacctgc

aggagatctttagtaatgagatggctaaggtggatgactctttcttccataggctggaggagtcctttttggtggaggaggataaaaagcacga
gcgccacccaatctttggcaatatcgtggacgaggtggcgtaccatgaaaagtacccaaccatatatcatctgaggaagaagcttgtagacag
tactgataaggctgacttgcggttgatctatctcgcgctggcgcatatgatcaaatttcggggacacttcctcatcgaggggggacctgaaccca
gacaacagcgatgtcgacaaactctttatccaactggttcagacttacaatcagcttttcgaagagaacccgatcaacgcatccggagttgacg
ccaaagcaatcctgagcgctaggctgtccaaatcccggcggctcgaaaacctcatcgcacagctccctggggagaagaagaacggcctgtt
tggtaatcttatcgccctgtccctcgggctgacccccaactttaaatctaacttcgacctggccgaagataccaagcttcaactgagcaaagaca
cctacgatgatgatctcgacaatctgctggcccagatcggcgaccagtacgcagacctttttttggcggcaaagaacctgtcagacgccattct
gctgagtgatattctgcgagtgaacacggagatcaccaaagctccgctgagcgctagtatgatcaagctctatgatgagcaccaccaagactt
gactttgctgaaggcccttgtcagacagcaactgcctgagaagtacaaggaaattttcttcgatcagtctaaaaatggctacgccggatacattg
acggcggagcaagccaggaggaattttacaaatttattaagcccatcttggaaaaaatggacggcaccgaggagctgctggtaaagcttaac
agagaagatctgttgcgcaaacagcgcactttcgacaatggaatcatcccccaccagattcacctgggcgaactgcacgctatcctcaggcg
gcaagaggatttctacccctttttgaaagataacagggaaaagattgagaaaatcctcacatttcggataccctactatgtaggcccccctcgccc
ggggaaattccagattcgcgtggatgactcgcaaatcagaagagaccatcactccctggaacttcgagaaagtcgtggataagggggcctct
gcccagtccttcatcgaaaggatgactaactttgataaaaatctgcctaacgaaaaggtgcttcctaaacactctctgctgtacgagtacttcaca
gtttataacgagctcaccaaggtcaaatacgtcacagaagggatgagaaagccagcattcctgtctggagatcagaagaaagctattgtggac
ctcctcttcaagacgaaccggaaagttaccgtgaaacagctcaaagaagactatttcaaaaagattgaatgtttcgactctgttgaaatcagcgg
agtggaggatcgcttcaacgcatccctgggaacgtatcacgatctcctgaaaatcattaaagacaaggacttcctggacaatgaggagaacg
aggacattcttgaggacattgtcctcacccttacgttgtttgaagatagggagatgattgaagaacgcttgaaaacttacgctcatctcttcgacg
acaaagtcatgaagcagctcaagaggcgccgatatacaggatgggggcggctgtcaagaaaactgatcaatgggatccgagacaagcaga
gtggaaagacaatcctggattttcttaagtccgatggatttgccaaccggaacttcattcagttgatccatgatgactctctcacctttaaggagga
catccagaaagcacaagtttctggccagggggacagtcttcacgagcacatcgctaatcttgcaggtagcccagctatcaaaaagggaatact
gcagaccgttaaggtcgtggatgaactcgtcaaagtaatgggaaggcataagcccgagaatatcgttatcgagatggcccgagagaaccaa
accacccagaagggacagaagaacagtagggaaaggatgaagaggattgaagagggtataaaagaactggggtcccaaatccttaagga
acacccagttgaaaacacccagcttcagaatgagaagctctacctgtactacctgcagaacggcagggacatgtacgtggatcaggaactgg
acatcaatcggctctccgactacgacgtggatcatatcgtgccccagtcttttctcaaagatgattctattgataataaagtgttgacaagatccga
taaaaacagagggaagagtgataacgtcccctcagaagaagttgtcaagaaaatgaaaaattattggcggcagctgctgaacgccaaactga
tcacacaacggaagttcgataatctgactaaggctgaacgaggtggcctgtctgagttggataaagccggtttcatcaaaaggcagcttgttga
gacacgccagatcaccaagcacgtggcccaaattctcgattcacgcatgaacaccaagtacgatgaaaatgacaaactgattcgagaggtga
aagttattactctgaagtctaagctggtctcagatttcagaaaggactttcagttttataaggtgagagagatcaacaattaccaccatgcgcatga
tgcctacctgaatgcagtggtaggcactgcacttatcaaaaaatatcccaagcttgaatctgaatttgtttacggagactataaagtgtacgatgtt
aggaaaatgatcgcaaagtctgagcaggaaataggcaaggccaccgctaagtacttcttttacagcaatattatgaatttttcaagaccgagat
tacactggccaatggagagattcggaagcgaccacttatcgaaacaaacggagaaacaggagaaatcgtgtgggacaagggtagggatttc
gcgacagtccggaaggtcctgtccatgccgcaggtgaacatcgttaaaaagaccgaagtacagaccggaggcttctccaaggaaagtatcc
tcccgaaaaggaacagcgacaagctgatcgcacgcaaaaaagattgggaccccaagaaatacggcggattcgattctcctacagtcgcttac
agtgtactggttgtggctaaagtggagaaagggaagtctaaaaaactcaaaagcgtcaaggaactgctgggcatcacaatcatggagcgatc
aagcttcgaaaaaaaccccatcgactttctcgaggcgaaaggatataaagaggtcaaaaaagacctcatcattaagcttcccaagtactctct
ttgagcttgaaaacggccggaaacgaatgctcgctagtgcgggcgtgctgcagaaaggtaacgagctggcactgccctctaaatacgttaatt
tcttgtatctggccagccactatgaaaagctcaaagggtctcccgaagataatgagcagaagcagctgttcgtggaacaacacaaacactacc
ttgatgagatcatcgagcaaataagcgaattctccaaaagagtgatcctcgccgacgctaacctcgataaggtgctttctgcttacaataagcac
agggataagcccatcagggagcaggcagaaaacattatccacttgtttactctgaccaacttgggcgcgcctgcagccttcaagtacttcgac
actaccatagacagaaagcggtacacctctacaaaggaggtcctggacgccacactgattcatcagtcaattacggggctctatgaaacaag
aatcgacctctctcagctcggtggagacgagggagctgataagcgcaccgccgatggttccgagttcgaaagccccaagaagaagaggaa
agtctaaccggtcatcatcaccatcaccattgagtttaaacccgctgatcagcctcgactgtgccttctagttgccagccatctgttgtttgcccct
cccccgtgccttccttgaccctggaaggtgccactcccactgtcctttcctaataaaatgaggaaattgcatcgcattgtctgagtaggtgtcattc

tattctggggggtggggtggggcaggacagcaagggggaggattgggaagacaatagcaggcatgctggggatgcggtgggctctatgg
cttctgaggcggaaagaaccagctggggctcgataccgtcgacctctagctagagcttggcgtaatcatggtcatagctgtttcctgtgtgaaat
tgttatccgctcacaattccacacaacatacgagccggaagcataaagtgtaaagcctagggtgcctaatgagtgagctaactcacattaattg
cgttgcgctcactgcccgctttccagtcgggaaacctgtcgtgccagctgcattaatgaatcggccaacgcgcggggagaggcggtttgcgt
attgggcgctcCtccgcttcctcgctcactgactcgctgcgctcggtcgttcggctgcggcgagcggtatcagctcactcaaaggcggtaata
cggttatccacagaatcaggggataacgcaggaaagaacatgtgagcaaaaggccagcaaaaggccaggaaccgtaaaaaggccgcgtt
gctggcgtttttccataggctccgcccccctgacgagcatcacaaaaatcgacgctcaagtcagaggtggcgaaacccgacaggactataaa
gataccaggcgtttccccctggaagctccctcgtgcgctctcctgttccgaccctgccgcttaccggatacctgtccgcctttctcccttcggga
agcgtggcgctttctcatagctcacgctgtaggtatctcagttcggtgtaggtcgttcgctccaagctgggctgtgtgcacgaaccccccgttca
gcccgaccgctgcgccttatccggtaactatcgtcttgagtccaacccggtaagacacgacttatcgccactggcagcagccactggtaacag
gattagcagagcgaggtatgtaggcggtgctacagagttcttgaagtggtggcctaactacggctacactagaagaacagtatttggtatctgc
gctctgctgaagccagttaccttcggaaaaagagttggtagctcttgatccggcaaacaaaccaccgctggtagcggtggtttttttgtttgcaa
gcagcagattacgcgcagaaaaaaaggatctcaagaagatcctttgatcttttctacggggtctgacgctcagtggaacgaaaactcacgttaa
gggattttggtcatgagattatcaaaaaggatcttcacctagatccttttaaattaaaaatgaagttttaaatcaatctaaagtatatatgagtaaactt
ggtctgacagttaccaatgcttaatcagtgaggcacctatctcagcgatctgtctatttcgttcatccatagttgcctgactccccgtcgtgtagata
actacgatacgggagggcttaccatctggccccagtgctgcaatgataccgcgagacccacgctcaccggctccagatttatcagcaataaa
ccagccagccggaagggccgagcgcagaagtggtcctgcaactttatccgcctccatccagtctattaattgttgccgggaagctagagtaa
gtagttcgccagttaatagtttgcgcaacgttgttgccattgctacaggcatcgtggtgtcacgctcgtcgtttggtatggcttcattcagctccggt
tcccaacgatcaaggcgagttacatgatcccccatgttgtgcaaaaaagcggttagctccttcggtcctccgatcgttgtcagaagtaagttggc
cgcagtgttatcactcatggttatggcagcactgcataattctcttactgtcatgccatccgtaagatgcttttctgtgactggtgagtactcaacca
agtcattctgagaatagtgtatgcggcgaccgagttgctcttgcccggcgtcaatacgggataataccgcgccacatagcagaactttaaaagt
gctcatcattggaaaacgttcttcggggcgaaaactctcaaggatcttaccgctgttgagatccagttcgatgtaacccactcgtgcacccaact
gatcttcagcatcttttactttcaccagcgtttctgggtgagcaaaaacaggaaggcaaaatgccgcaaaaaagggaataagggcgacacgg
aaatgttgaatactcatactcttcctttttcaatattattgaagcatttatcagggttattgtctcatgagcggatacatatttgaatgtatttagaaaaat
aaacaaataggggttccgcgcacatttccccgaaaagtgccacctgacgtcgacggatcgggagatcgatctcccgatcccctagggtcgac
tctcagtacaatctgctctgatgccgcatagttaagccagtatctgctccctgcttgtgtgttggaggtcgctgagtagtgcgcgagcaaaattta
agctacaacaaggcaaggcttgaccgacaattgcatgaagaatctgcttagggttaggcgttttgcgctgcttcgcgatgtac

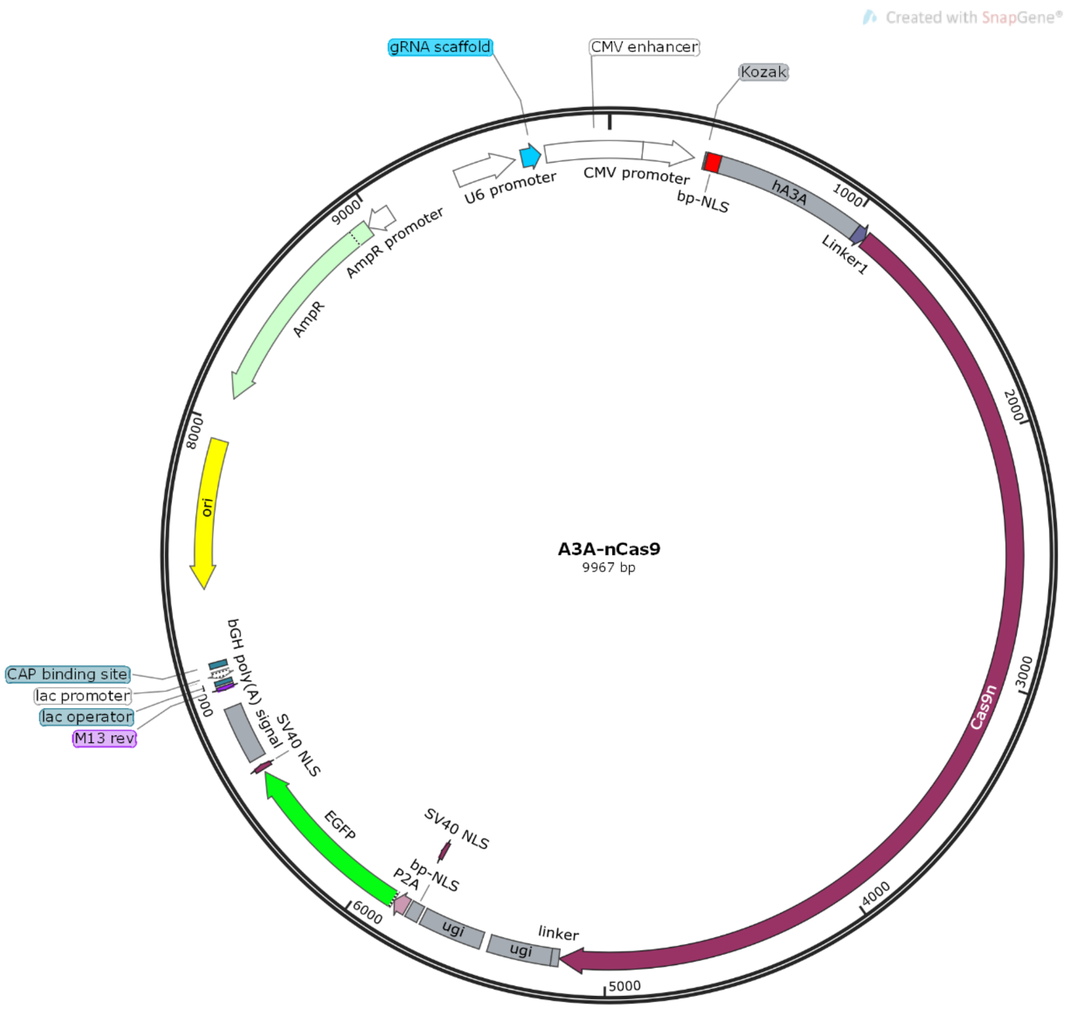

Supplementary Fig. 13 APOBEC3A-nCas9 plasmid map

APOBEC3A-nCas9 plasmid sequence:

atatgccaagtacgccccctattgacgtcaatgacggtaaatggcccgcctggcattatgcccagtacatgaccttatgggactttcctacttgg
cagtacatctacgtattagtcatcgctattaccatggtgatgcggttttggcagtacatcaatgggcgtggatagcggtttgactcacggggattt
ccaagtctccaccccattgacgtcaatgggagtttgttttggcaccaaaatcaacgggactttccaaaatgtcgtaacaactccgccccattgac
gcaaatgggcggtaggcgtgtacggtgggaggtctatataagcagagctggtttagtgaaccgtcagatccgctagagatccgcggccgcc
accatgaagaggaccgccgatggctctgagttcgagagccccaagaagaagcggaaggtggaggcatctccagcaagcggaccaaggc
acctgatggacccccacatcttcacctctaactttaacaatggcatcggcaggcacaagacatacctgtgctatgaggtggagcgcctggaca
acggcaccagcgtgaagatggatcagcacagaggcttcctgcacaaccaggccaagaatctgctgtgcggcttctacggccggcacgcag
agctgagatttctggacctggtgcctagcctgcagctggatccagcccagatctatagggtgacctggttcatcagctggtccccatgcttttcct
ggggatgtgcaggagaggtgcgcgccttcctgcaggagaatacacacgtgcggctgagaatctttgccgcccggatctacgactatgatcct
ctgtacaaggaggccctgcagatgctgagagacgcaggagcccaggtgtccatcatgacctatgatgagttcaagcactgctgggacacatt
tgtggatcaccagggctgtccctttcagccttgggacggactggatgagcactcccaggccctgtctggcaggctgagggccatcctgcaga
accagggcaatagcggcagcgagactcccgggacctcagagtccgccacacccgaaagtgacaagaagtacagcatcggcctggccatc
ggcaccaactctgtgggctgggccgtgatcaccgacgagtacaaggtgcccagcaagaaattcaaggtgctgggcaacaccgaccggcac
agcatcaagaagaacctgatcggagccctgctgttcgacagcggcgaaacagccgaggccacccggctgaagagaaccgccagaagaa
gatacaccagacggaagaaccggatctgctatctgcaagagatcttcagcaacgagatggccaaggtggacgacagcttcttccacagactg
gaagagtccttcctggtggaagaggataagaagcacgagcggcaccccatcttcggcaacatcgtggacgaggtggcctaccacgagaag
taccccaccatctaccacctgagaaagaaactggtggacagcaccgacaaggccgacctgcggctgatctatctggccctggcccacatgat

caagttccggggccacttcctgatcgagggcgacctgaaccccgacaacagcgacgtggacaagctgttcatccagctggtgcagacctac
aaccagctgttcgaggaaaaccccatcaacgccagcggcgtggacgccaaggccatcctgtctgccagactgagcaagagcagacggct
ggaaaatctgatcgcccagctgcccggcgagaagaagaatggcctgttcggaaacctgattgccctgagcctgggcctgacccccaacttc
aagagcaacttcgacctggccgaggatgccaaactgcagctgagcaaggacacctacgacgacgacctggacaacctgctggcccagatc
ggcgaccagtacgccgacctgtttctggccgccaagaacctgtccgacgccatcctgctgagcgacatcctgagagtgaacaccgagatca
ccaaggcccccctgagcgcctctatgatcaagagatacgacgagcaccaccaggacctgaccctgctgaaagctctcgtgcggcagcagct
gcctgagaagtacaaagagattttcttcgaccagagcaagaacggctacgccggctacattgacggcggagccagccaggaagagttctac
aagttcatcaagcccatcctggaaaagatggacggcaccgaggaactgctcgtgaagctgaacagagaggacctgctgcggaagcagcg
gaccttcgacaacggcagcatccccccaccagatccacctgggagagctgcacgccattctgcggcggcaggaagatttttacccattcctga
aggacaaccgggaaaagatcgagaagatcctgaccttccgcatcccctactacgtgggccctctggccaggggaaacagcagattcgcctg
gatgaccagaaagagcgaggaaaccatcaccccctggaacttcgaggaagtggtggacaagggcgcttccgcccagagcttcatcgagcg
gatgaccaacttcgataagaacctgcccaacgagaaggtgctgcccaagcacagcctgctgtacgagtacttcaccgtgtataacgagctga
ccaaagtgaaatacgtgaccgagggaatgagaaagcccgccttcctgagcggcgagcagaaaaaggccatcgtggacctgctgttcaaga
ccaaccggaaagtgaccgtgaagcagctgaaagaggactacttcaagaaaatcgagtgcttcgactccgtggaaatctccggcgtggaaga
tcggttcaacgcctccctgggcacataccacgatctgctgaaaattatcaaggacaaggacttcctggacaatgaggaaaacgaggacattct
ggaagatatcgtgctgaccctgacactgtttgaggacagagagatgatcgaggaacggctgaaaacctatgcccacctgttcgacgacaaag
tgatgaagcagctgaagcggcggagatacaccggctggggcaggctgagccggaagctgatcaacggcatccgggacaagcagtccgg
caagacaatcctggatttcctgaagtccgacggcttcgccaacagaaacttcatgcagctgatccacgacgacagcctgacctttaaagagga
catccagaaagcccaggtgtccggccagggcgatagcctgcacgagcacattgccaatctggccggcagccccgccattaagaagggcat
cctgcagacagtgaaggtggtggacgagctcgtgaaagtgatgggccggcacaagcccgagaacatcgtgatcgaaatggccagagaga
accagaccacccagaagggacagaagaacagccgcgagagaatgaagcggatcgaagagggcatcaaagagctgggcagccagatcc
tgaaagaacaccccgtggaaaacacccagctgcagaacgagaagctgtacctgtactacctgcagaatgggcgggatatgtacgtggacca
ggaactggacatcaaccggctgtccgactacgatgtggaccatatcgtgcctcagagctttctgaaggacgactccatcgacaacaaggtgct
gaccagaagcgacaagaaccggggcaagagcgacaacgtgccctccgaagaggtcgtgaagaagatgaagaactactggcggcagctg
ctgaacgccaagctgattacccagagaaagttcgacaatctgaccaaggccgagagaggcggcctgagcgaactggataaggccggcttc
atcaagagacagctggtggaaacccggcagatcacaaagcacgtggcacagatcctggactcccggatgaacactaagtacgacgagaat
gacaagctgatccgggaagtgaaagtgatcaccctgaagtccaagctggtgtccgatttccggaaggatttccagttttacaaagtgcgcgag
atcaacaactaccaccacgcccacgacgcctacctaaacgccgtcgtgggaaccgccctgatcaaaaagtaccctaagctggaaagcgagt
tcgtgtacggcgactacaaggtgtacgacgtgcggaagatgatcgccaagagcgagcaggaaatcggcaaggctaccgccaagtacttctt
ctacagcaacatcatgaactttttcaagaccgagattaccctggccaacggcgagatccggaagcggcctctgatcgagacaaacggcgaa
accggggagatcgtgtgggataagggccgggattttgccaccgtgcggaaagtgctgagcatgccccaagtgaatatcgtgaaaaagaccg
aggtgcagacaggcggcttcagcaaagagtctatcctgcccaagaggaacagcgataagctgatcgccagaaagaaggactgggacccta
agaagtacggcggcttcgacagccccaccgtggcctattctgtgctggtggtggccaaagtggaaaagggcaagtccaagaaactgaaga
gtgtgaaagagctgctggggatcaccatcatggaaagaagcagcttcgagaagaatcccatcgactttctggaagccaagggctacaaaga
agtgaaaaaggacctgatcatcaagctgcctaagtactccctgttcgagctggaaaacggccggaagagaatgctggcctctgccggcgaa
ctgcagaagggaaacgaactggccctgccctccaaatatgtgaacttcctgtacctggccagccactatgagaagctgaagggctcccccga
ggataatgagcagaaacagctgtttgtggaacagcacaagcactacctggacgagatcatcgagcagatcagcgagttctccaagagagtg
atcctggccgacgctaatctggacaaagtgctgtccgcctacaacaagcaccgggataagcccatcagagagcaggccgagaatatcatcc
acctgtttaccctgaccaatctgggagcccctgccgccttcaagtactttgacaccaccatcgaccggaagaggtacaccagcaccaaagag
gtgctggacgccaccctgatccaccagagcatcaccggcctgtacgagacacggatcgacctgtctcagctgggaggtgacagcggcggg
agcggcgggagcgggggggagcactaatctgagcgacatcattgagaaggagactgggaaacagctggtcattcaggagtccatcctgatg
ctgcctgaggaggtggaggaagtgatcggcaacaagccagagtctgacatcctggtgcacaccgcctacgacgagtccacagatgagaat
gtgatgctgctgacctctgacgcccccgagtataagccttgggccctggtcatccaggattctaacggcgagaataagatcaagatgctgagc
ggaggatccggaggatctggaggcagcaccaacctgtctgacatcatcgagaaggagacaggcaagcagctggtcatccaggagagcat

cctgatgctgcccgaagaagtcgaagaagtgatcggaaacaagcctgagagcgatatcctggtccataccgcctacgacgagagtaccgac
gaaaatgtgatgctgctgacatccgacgccccagagtataagccctgggctctggtcatccaggattccaacggagagaacaaaatcaaaat
gctgtctggcggctcaaaaagaaccgccgacggcagcgaattcgagcccaagaagaagaggaaagtcggaagcggagctactaacttca
gcctgctgaagcaggctggagacgtggaggagaaccctggacctatggtgagcaagggcgaggagctgttcaccggggtggtgcccatc
ctggtcgagctggacggcgacgtaaacggccacaagttcagcgtgtccggcgagggcgagggcgatgccacctacggcaagctgaccct
gaagttcatctgcaccaccggcaagctgcccgtgccctggcccaccctcgtgaccaccctgacctatggagtgcagtgcttcagccgctacc
ccgaccacatgaagcagcacgacttcttcaagtccgccatgcccgaaggctacgtccaggagcgcaccatcttcttcaaggacgacggcaa
ctacaagacccgcgccgaggtgaagttcgagggcgacaccctggtgaaccgcatcgagctgaagggcatcgacttcaaggaggacggca
acatcctggggcacaagctggagtacaactacaacagccacaacgtctatatcatggccgacaagcagaagaacggcatcaaggtgaactt
caagatccgccacaacatcgaggacggcagcgtgcagctcgccgaccactaccagcagaacacccccatcggcgacggccccgtgctgc
tgcccgacaaccactacctgagcacccagtccgccctgagcaaagaccccaacgagaagcgcgatcacatggtcctgctggagttcgtgac
cgccgccgggatcactctcggcatggacgagctgtacaagtctggtggttctcccaagaagaaaaggaaagtctaagtttaaacccgctgatc
agcctcgactgtgccttctagttgccagccatctgttgtttgcccctcccccgtgccttccttgaccctggaaggtgccactcccactgtcctttcc
taataaaatgaggaaattgcatcgcattgtctgagtaggtgtcattctattctggggggtggggtggggcaggacagcaagggggaggattgg
gaagagaatagcaggcatgctggggatgcggtgggctctatggcttctgaggcggaaagaaccagctggggctcgataccgtcgacctcta
gctagagcttggcgtaatcatggtcatagctgtttcctgtgtgaaattgttatccgctcacaattccacacaacatacgagccggaagcataaagt
gtaaagcctagggtgcctaatgagtgagctaactcacattaattgcgttgcgctcactgcccgctttccagtcgggaaacctgtcgtgccagct
gcattaatgaatcggccaacgcgcggggagaggcggtttgcgtattgggcgctcttccgcttcctcgctcactgactcgctgcgctcggtcgtt
cggctgcggcgagcggtatcagctcactcaaaggcggtaatacggttatccacagaatcaggggataacgcaggaaagaacatgtgagca
aaaggccagcaaaaggccaggaaccgtaaaaaggccgcgttgctggcgtttttccataggctccgcccccctgacgagcatcacaaaaatc
gacgctcaagtcagaggtggcgaaacccgacaggactataaagataccaggcgtttccccctggaagctccctcgtgcgctctcctgttccg
accctgccgcttaccggatacctgtccgcctttctcccttcgggaagcgtggcgctttctcatagctcacgctgtaggtatctcagttcggtgtag
gtcgttcgctccaagctgggctgtgtgcacgaaccccccgttcagcccgaccgctgcgccttatccggtaactatcgtcttgagtccaacccg
gtaagacacgacttatcgccactggcagcagccactggtaacaggattagcagagcgaggtatgtaggcggtgctacagagttcttgaagtg
gtggcctaactacggctacactagaagaacagtatttggtatctgcgctctgctgaagccagttaccttcggaaaaagagttggtagctcttgat
ccggcaaacaaaccaccgctggtagcggtggtttttttgtttgcaagcagcagattacgcgcagaaaaaaaggatctcaagaagatcctttgat
cttttctacggggtctgacgctcagtggaacgaaaactcacgttaagggattttggtcatgagattatcaaaaaggatcttcacctagatcctttta
aattaaaaatgaagttttaaatcaatctaaagtatatatgagtaaacttggtctgacagttaccaatgcttaatcagtgaggcacctatctcagcgat
ctgtctatttcgttcatccatagttgcctgactccccgtcgtgtagataactacgatacgggagggcttaccatctggccccagtgctgcaatgat
accgcgagacccacgctcaccggctccagatttatcagcaataaaccagccagccggaagggccgagcgcagaagtggtcctgcaacttta
tccgcctccatccagtctattaattgttgccgggaagctagagtaagtagttcgccagttaatagtttgcgcaacgttgttgccattgctacaggca
tcgtggtgtcacgctcgtcgtttggtatggcttcattcagctccggttcccaacgatcaaggcgagttacatgatcccccatgttgtgcaaaaaag
cggttagctccttcggtcctccgatcgttgtcagaagtaagttggccgcagtgttatcactcatggttatggcagcactgcataattctcttactgtc
atgccatccgtaagatgcttttctgtgactggtgagtactcaaccaagtcattctgagaatagtgtatgcggcgaccgagttgctcttgcccggc
gtcaatacgggataataccgcgccacatagcagaactttaaaagtgctcatcattggaaaacgttcttcggggcgaaaactctcaaggatctta
ccgctgttgagatccagttcgatgtaacccactcgtgcacccaactgatcttcagcatcttttactttcaccagcgtttctgggtgagcaaaaaca
ggaaggcaaaatgccgcaaaaaagggaataagggcgacacggaaatgttgaatactcatactcttcctttttcaatattattgaagcatttatca
gggttattgtctcatgagcggatacatatttgaatgtatttagaaaaataaacaaataggggttccgcgcacatttccccgaaaagtgccacctga
cgtcgacggatcgggagatcgatctcccgatcccctagggtcgactctcagtacaatctgctctgatgccgcatagttaagccagtatctgctc
cctgcttgtgtgttggaggtcgctgagtagtgcgcgagcaaaatttaagctacaacaaggcaaggcttgaccgacaattgcatgaagaatctg
cttagggttaggcgttttgcgctgcttcgcgatgtacgggccagatatacgcgtggatccctgcagttaattaaagagggcctatttcccatgatt
ccttcatatttgcatatacgatacaaggctgttagagagataattagaattaatttgactgtaaacacaaagatattagtacaaaatacgtgacgta
gaaagtaataatttcttgggtagtttgcagttttaaaattatgttttaaaatggactatcatatgcttaccgtaacttgaaagtatttcgatttcttggcttt
atatatcttgtggaaaggacgaaacaccgggtcttcgagaagacctgttttagagctagaaatagcaagttaaaataaggctagtccgttatcaa

cttgaaaaagtggcaccgagtcggtgctttttttatgcatggtaccgacattgattattgactagttattaatagtaatcaattacggggtcattagtt catagcccatatatggagttccgcgttacataacttacggtaaatggcccgcctggctgaccgcccaacgacccccgcccattgacgtcaata atgacgtatgttcccatagtaacgccaatagggactttccattgacgtcaatgggtggagtatttacggtaaactgcccacttggcagtacatcaa gtgtatc
