# Supplementary material for: Automated high-throughput genome editing platform with an AI learning in situ prediction model
Source: Nat Commun. 2022 Nov 30;13:7386. doi: 10.1038/s41467-022-35056-0 (PMC9712529; doi:10.1038/s41467-022-35056-0)
Supplement: Supplementary file 2 — Description of Additional Supplementary Files [file 41467_2022_35056_MOESM2_ESM.pdf]

**Title: Supplementary Data 1.**

**Description:** The sequences of the 1210 genes and protospacers.

**Title: Supplementary Data 2.**

**Description:** The 1210 corresponding primer pairs for construction of gRNA plasmids.

**Title: Supplementary Data 3.**

**Description:** Three pairs of primers for the analysis of cells editing results.

**Title: Supplementary Data 4.**

**Description:** The sample sites for another round of new colonies picking of gRNA plasmids verification**.**

**Title: Supplementary Data 5.**

**Description:** The sample sites of correct assembly for gRNA plasmids extraction.

**Title: Supplementary Data 6.**

**Description:** The sample sites for preparing a new round of PCR.

**Title: Supplementary Data 7.**

**Description:** The new primer pairs for a new round of PCR.

**Title: Supplementary Data 8.**

**Description:** The editing efficiencies results of correct samples of PCR.

**Title: Supplementary Data 9.**

**Description:** Editing efficiencies by BE4max and chromatin accessibility data of 1210 targets.

**Title: Supplementary Data 10.**

**Description:** The editing results of Anc-BE4max_HEK293T, hyA3A-BE4max_HEK293T, BE4max_HepG2, Anc-BE4max_HepG2 and hyA3A-BE4max_HepG2
