# Supplementary material for: Automated high-throughput genome editing platform with an AI learning in situ prediction model
Source: Nat Commun. 2022 Nov 30;13:7386. doi: 10.1038/s41467-022-35056-0 (PMC9712529; doi:10.1038/s41467-022-35056-0)
Supplement: Supplementary file 3 — Reporting Summary [file 41467_2022_35056_MOESM3_ESM.pdf]

nature portfolio

Corresponding author(s): Changhao Bi, Xueli Zhang, Meng Wang

Last updated by author(s): Nov 13, 2022

Reporting Summary

Nature Portfolio wishes to improve the reproducibility of the work that we publish. This form provides structure for consistency and transparency in reporting. For further information on Nature Portfolio policies, see our Editorial Policies and the Editorial Policy Checklist.

Statistics

For all statistical analyses, confirm that the following items are present in the figure legend, table legend, main text, or Methods section.

n/a	Confirmed	
☐	☒	The exact sample size (*n*) for each experimental group/condition, given as a discrete number and unit of measurement
☐	☒	A statement on whether measurements were taken from distinct samples or whether the same sample was measured repeatedly
☐	☒	The statistical test(s) used AND whether they are one- or two-sided<br>*Only common tests should be described solely by name; describe more complex techniques in the Methods section.*
☒	☐	A description of all covariates tested
☒	☐	A description of any assumptions or corrections, such as tests of normality and adjustment for multiple comparisons
☐	☒	A full description of the statistical parameters including central tendency (e.g. means) or other basic estimates (e.g. regression coefficient) AND variation (e.g. standard deviation) or associated estimates of uncertainty (e.g. confidence intervals)
☐	☒	For null hypothesis testing, the test statistic (e.g. *F*, *t*, *r*) with confidence intervals, effect sizes, degrees of freedom and *P* value noted<br>*Give P values as exact values whenever suitable.*
☒	☐	For Bayesian analysis, information on the choice of priors and Markov chain Monte Carlo settings
☒	☐	For hierarchical and complex designs, identification of the appropriate level for tests and full reporting of outcomes
☒	☐	Estimates of effect sizes (e.g. Cohen's *d*, Pearson's *r*), indicating how they were calculated

Our web collection on statistics for biologists contains articles on many of the points above.

Software and code

Policy information about availability of computer code

Data collection	Sanger Sequencing by tsingke
Data analysis	EditR(http://baseeditr.com/)

For manuscripts utilizing custom algorithms or software that are central to the research but not yet described in published literature, software must be made available to editors and reviewers. We strongly encourage code deposition in a community repository (e.g. GitHub). See the Nature Portfolio guidelines for submitting code & software for further information.

Data

Policy information about availability of data

All manuscripts must include a data availability statement. This statement should provide the following information, where applicable:
- Accession codes, unique identifiers, or web links for publicly available datasets
- A description of any restrictions on data availability
- For clinical datasets or third party data, please ensure that the statement adheres to our policy

There is no restriction on data associated with this study. Pasmids used in this work are available on Addgene.

Human research participants

Policy information about studies involving human research participants and Sex and Gender in Research.

Reporting on sex and gender	It is not refered
Population characteristics	It is not refered
Recruitment	It is not refered
Ethics oversight	It is not refered

Note that full information on the approval of the study protocol must also be provided in the manuscript.

Field-specific reporting

Please select the one below that is the best fit for your research. If you are not sure, read the appropriate sections before making your selection.

- [x] Life sciences
- [ ] Behavioural & social sciences
- [ ] Ecological, evolutionary & environmental sciences

For a reference copy of the document with all sections, see nature.com/documents/nr-reporting-summary-flat.pdf

Life sciences study design

All studies must disclose on these points even when the disclosure is negative.

Sample size	No statistical methods were used to predetermine sample size for experiments.
Data exclusions	No data was excluded from data analysis, and all data obtained was used in the statistic analysis.
Replication	Data was obtained in the fashion of biological replicates. All attempts for replication were successful.
Randomization	For the HEK293T base editing, all the samples were collected for Sanger sequencing.
Blinding	For the HEK293T base editing, all the samples were collected for Sanger sequencing.

Reporting for specific materials, systems and methods

We require information from authors about some types of materials, experimental systems and methods used in many studies. Here, indicate whether each material, system or method listed is relevant to your study. If you are not sure if a list item applies to your research, read the appropriate section before selecting a response.

Materials & experimental systems

n/a	Involved in the study	
x		Antibodies
	x	Eukaryotic cell lines
x		Palaeontology and archaeology
x		Animals and other organisms
x		Clinical data
x		Dual use research of concern

Methods

n/a	Involved in the study	
x		ChIP-seq
	x	Flow cytometry
x		MRI-based neuroimaging

Eukaryotic cell lines

Policy information about cell lines and Sex and Gender in Research

Cell line source(s)	HEK 293T from ATCC
Authentication	No cell lines were authenticated
Mycoplasma contamination	Cells were not tested for mycoplasma contamination.
Commonly misidentified lines (See ICLAC register)	No cell lines were used that are in the ICLAC register.

Flow Cytometry

Plots

Confirm that:

- [x] The axis labels state the marker and fluorochrome used (e.g. CD4-FITC).
- [x] The axis scales are clearly visible. Include numbers along axes only for bottom left plot of group (a 'group' is an analysis of identical markers).
- [x] All plots are contour plots with outliers or pseudocolor plots.
- [x] A numerical value for number of cells or percentage (with statistics) is provided.

Methodology

Sample preparation	HEK293T cells were harvested, and single cells were sorted according to the GFP fluorescence generated due to the reporter gene carried by the BE4max plasmid.
Instrument	MoFlo XDP high-speed sorter, Beckman Coulter, Fullerton, CA, USA
Software	MoFlo XDP Summit (v 5.2)
Cell population abundance	GFP positive single cells were sorted into 96-well plates and cells were expanded for sequencing
Gating strategy	Samples was analyzed by flow cytometry with the following parameters: FSC (voltage 180 V), and SSC (voltage 280 V), the threshold was set as 5%(triggered on FSC channel).In the figure of fsc-a versus fsc-w, remove the adherent cells with large fsc-w value, and sort GFP positive cells of FL1(voltage 450 V, excitation at 488 nm, emission fluorescence at 529 ± 14 nm) versus FL4(voltage 450 V, excitation at 488 nm, emission fluorescence at 670 ± 30 nm), in order to remove the dead cells on the diagonal.

- [ ] Tick this box to confirm that a figure exemplifying the gating strategy is provided in the Supplementary Information.
